# Influência da Obesidade na Segurança e Eficácia do Tratamento Antitrombótico: Uma Revisão Sistemática e Metanálise

**DOI:** 10.36660/abc.20240544

**Published:** 2025-05-12

**Authors:** Beatriz Rocha Darzé, Queila Oliveira Borges, Mateus S. Viana, Eduardo Sahade Darzé, Luiz Eduardo Fonteles Ritt

**Affiliations:** 1 Escola Bahiana de Medicina e Saúde Pública Salvador BA Brasil Escola Bahiana de Medicina e Saúde Pública, Salvador, BA – Brasil; 2 D’Or Research Institute Hospital Cardio Pulmonar Salvador BA Brasil D’Or Research Institute, IDOR, Hospital Cardio Pulmonar, Salvador, BA – Brasil

**Keywords:** Síndrome Coronariana Aguda, Fibrinolíticos, Obesidade, Prognóstico, Tromboembolia Venosa

## Abstract

**Fundamento:**

Indivíduos obesos têm sido historicamente sub-representados em ensaios clínicos. Considerando sua associação com maior risco de tromboembolismo venoso (TEV) e síndrome coronariana aguda (SCA), é necessário estabelecer um regime de anticoagulação mais adequado para esse grupo de pacientes.

**Objetivos:**

Avaliar a influência da obesidade na segurança e eficácia do tratamento antitrombótico em pacientes com SCA ou TEV.

**Métodos:**

Trata-se de uma revisão sistemática e metanálise que utilizou 5 principais bases de dados internacionais. Selecionamos ensaios clínicos ou estudos observacionais que compararam a ocorrência de desfechos clínicos (mortalidade ou sangramento) entre pacientes obesos e não obesos em uso de anticoagulantes parenterais para tratamento de SCA ou TEV. Foram considerados valores de p < 0,05 para todas as análises.

**Resultados:**

Foram elegíveis 6 artigos, incluindo um total de 40.939 pacientes, sendo 3 ensaios clínicos randomizados e 3 coortes retrospectivas. Dos pacientes, 87,7% apresentaram SCA. A incidência de sangramento maior foi semelhante entre os grupos (risco relativo [RR]: 0,90, intervalo de confiança [IC] de 95%: 0,77 a 1,04, p = 0,14). O desfecho permaneceu comparável quando os estudos foram analisados separadamente por anticoagulante: enoxaparina (RR: 0,87, IC 95%, 0,70 a 1,08, p = 0,21) ou heparina não fracionada (RR: 0,96, IC 95%, 0,79 a 1,17, p = 0,67). A taxa de mortalidade foi medida em apenas 2 estudos, ambos sobre SCA, e foi menor em pacientes obesos (RR: 0,71, IC 95% 0,59 a 0,87, p = 0,0007).

**Conclusão:**

Em pacientes tratados para TEV ou SCA, as taxas de sangramento foram comparáveis entre obesos e não obesos, independentemente do anticoagulante usado. A menor taxa de mortalidade observada em pacientes obesos pode representar o efeito de fatores de confusão não contabilizados.

## Graphical abstract

**Figura Central:**
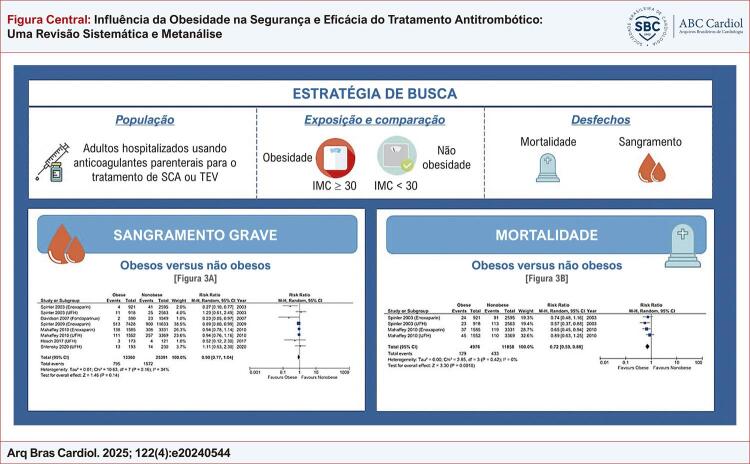
Influência da Obesidade na Segurança e Eficácia do Tratamento Antitrombótico:

IMC: índice de massa corporal; SCA: síndrome coronariana aguda; TEV: tromboembolismo venoso.

## Introdução

A obesidade é uma doença crônica, que atinge níveis epidêmicos.^1^ Segundo a Organização Mundial da Saúde, a prevalência da obesidade quase triplicou nos últimos 50 anos.^2^

O tratamento antitrombótico é um pilar no tratamento da síndrome coronariana aguda (SCA) e do tromboembolismo venoso (TEV). Ele reduz significativamente a ocorrência de eventos isquêmicos, mas aumenta o risco de sangramento, o que está relacionado a uma maior incidência de morte, infarto agudo do miocárdio e acidente vascular cerebral.^3^ Os anticoagulantes parenterais são administrados com titulação de dose com base no peso dos pacientes,^4^ e o limite máximo da dose para pacientes obesos não é bem estudado. Um estudo com enoxaparina demonstrou que a população obesa tem maior incidência de subdosagem e, entre aqueles que recebem a dose recomendada, os pacientes obesos têm maior risco de sangramento.^5^

Por outro lado, o fondaparinux, quando usado para o tratamento de SCA, é usado em dose fixa.^4^ Os estudos OASIS-5 e OASIS-6 mostraram que o fondaparinux é não inferior à enoxaparina para o composto de eventos isquêmicos e está associado a menor mortalidade, provavelmente devido à redução de eventos hemorrágicos.^6,7^ No entanto, como a população obesa não foi adequadamente representada nesses estudos, a aplicação desses resultados a pacientes com índice de massa corporal (IMC) ≥ 30 kg/m^2^ é incerta.

O excesso de gordura corporal ainda pode afetar a farmacocinética e a farmacodinâmica dos medicamentos,^8^ o que, combinado ao fato da população obesa ser historicamente sub-representada em ensaios clínicos, leva a incertezas quanto ao real efeito da obesidade na eficácia e segurança do tratamento antitrombótico.

O objetivo do presente estudo foi avaliar a associação entre a obesidade e a ocorrência de desfechos clínicos entre pacientes tratados com anticoagulação parenteral para SCA ou TEV.

## Métodos

### Desenho do estudo

Trata-se de uma revisão sistemática da literatura com metanálise.

### Estratégia de busca

MEDLINE/PubMed, SciELO, EMBASE, Lilacs e Cochrane Library (CENTRAL), foram sistematicamente pesquisados entre 9 de março de 2021 e 12 de março de 2021. A identificação dos estudos foi baseada na estratégia de busca PECO, que significa população (P), exposição (E), comparação (C) e desfechos (O, do inglês, *outcomes*), que foram definidos da seguinte forma:

P: Adultos hospitalizados em uso de anticoagulantes parenterais (heparina não fracionada [HNF], heparina de baixo peso molecular [HBPM], fondaparinux ou bivalirudina) para o tratamento de SCA ou TEV. E: Obesidade (≥ 30 kg/m^2^) C: Não obesidade (< 30 kg/m^2^) O: Eventos cardíacos adversos maiores (MACE) ou sangramento

As ferramentas PubMed, MeSH e DeCS foram usadas conforme apropriado para cada base de dados. Os detalhes das estratégias de busca realizadas para cada base de dados estão disponíveis nos Métodos Suplementares.

Além disso, foi realizada uma busca manual nas referências bibliográficas dos estudos selecionados.

### Critérios de inclusão

#### Tipos de estudo

Foram incluídos estudos originais publicados em qualquer ano, com texto completo em inglês, português ou espanhol. Apenas artigos caracterizados como ensaios clínicos randomizados, estudos de coorte prospectivos, estudos de coorte retrospectivos e estudos de caso-controle foram incluídos.

#### População

Selecionamos artigos que incluíram pacientes hospitalizados com diagnóstico de SCA (angina instável, infarto do miocárdio sem supradesnivelamento do segmento ST [NSTEMI] e infarto do miocárdio com supradesnivelamento do segmento ST [STEMI]) ou TEV tratados com terapia anticoagulante parenteral (HNF, HBPM, fondaparinux ou bivalirudina), considerando obesidade como exposição e não obesidade como comparador.

#### Desfechos clínicos

Os desfechos clínicos incluíram a ocorrência de MACE (definidos como óbito, infarto do miocárdio ou acidente vascular cerebral) ou sangramento maior. A definição de cada desfecho para nosso estudo foi baseada nas definições adotadas pelos artigos incluídos. As definições de sangramento maior adotadas em cada estudo estão disponíveis na Tabela Suplementar 1.

## Critérios de exclusão

### Tipos de estudo

Foram excluídos relatos de caso, séries de casos, revisões de literatura e revisões sistemáticas. Estudos de prevenção/profilaxia também foram excluídos.

### População

Estudos que incluíram pacientes menores de 18 anos e adultos com depuração de creatinina < 10 mL/min foram excluídos.

## Identificação e seleção dos estudos

Dois autores realizaram a busca, seleção e aplicação dos critérios de elegibilidade de forma independente.

Com base nos resultados das buscas realizadas nas bases de dados, o processo de seleção foi realizado individualmente nas seguintes 3 etapas: (1) remoção de duplicatas; (2) exclusão de artigos que não atendiam aos critérios de elegibilidade com base no título e resumo; (3) e leitura completa dos artigos selecionados, com nova aplicação dos critérios de elegibilidade para identificar sua qualidade e relevância para o objetivo proposto. Quaisquer divergências entre os autores foram resolvidas por meio de discussão e diálogo na presença de um terceiro autor.

A ferramenta Rayyan QCRI foi usada para seleção dos artigos, e ambos os pesquisadores foram cegados para as decisões um do outro durante todo o processo.

## Extração de dados

As seguintes características dos estudos foram extraídas: título, referência, tipo de estudo (ensaio clínico randomizado ou estudo observacional), país, ano de publicação e tamanho da amostra. Os seguintes dados foram coletados sobre os participantes de cada estudo: número de participantes, idade, número de participantes do sexo masculino e feminino, número de participantes obesos e não obesos, IMC médio, tipo de SCA (STEMI, NSTEMI ou angina instável) e TEV (trombose venosa profunda ou embolia pulmonar) e estratégia antitrombótica realizada.

Foram extraídos todos os desfechos avaliados pelos estudos, com foco nos desfechos clínicos e na comparação entre indivíduos obesos e não obesos.

O processo de extração de dados foi realizado de forma independente por dois autores, e as discrepâncias foram resolvidas por discussão com um terceiro autor. O software Review Manager (Revman), versão 5.4 foi usado para registro, extração e gerenciamento dos dados.

## Análise de risco de viés

A avaliação do risco de viés incluiu a avaliação dos métodos de randomização, alocação de tratamento, cegamento, seleção e comparabilidade de grupos de estudo e avaliação dos desfechos, sendo realizada no nível dos estudos. Foram usadas as seguintes ferramentas: a Escala Newcastle-Ottawa (NOS) para estudos observacionais e a Ferramenta de Risco de Viés Cochrane (RoB) para ensaios clínicos randomizados. Essas avaliações foram realizadas por dois autores independentes, e as discrepâncias foram resolvidas por discussão com um terceiro autor.

O viés de publicação foi avaliado por meio de inspeção visual de gráficos de funil correspondentes às metanálises de desfechos primários, que foram gerados usando o software RevMan 5.4.

## Análise da qualidade da evidência

Para avaliar a qualidade da evidência, a metodologia GRADE foi aplicada a cada desfecho estudado. A avaliação foi realizada por dois autores independentes, e as discrepâncias foram resolvidas por discussão com um terceiro autor.

## Análise estatística

Para comparação dos desfechos primários entre os grupos de pacientes obesos e não obesos, o risco relativo (RR) e os intervalos de confiança (IC) de 95% foram usados como parâmetros analíticos. Para cada grupo antitrombótico (enoxaparina, HNF, fondaparinux), o RR e o IC de 95% foram gerados a partir do número absoluto de pacientes e de desfechos. A análise estatística e os gráficos de floresta foram realizados usando o software RevMan 5.4.

A heterogeneidade foi avaliada pela inspeção visual dos gráficos de floresta (análise), juntamente com a consideração do teste qui-quadrado para heterogeneidade e a estatística I^2^ (teste de Higgins). A heterogeneidade foi considerada estatisticamente significativa com p < 0,1 ou pela porcentagem de heterogeneidade, que foi classificada como baixa (25% ou menos), moderada (26% a 50%) ou alta (maior que 50%). Os estudos também foram examinados quanto à heterogeneidade metodológica e clínica, particularmente quando identificada heterogeneidade estatisticamente significativa.

Os resultados de grupos comparáveis de estudos foram agrupados usando um modelo de efeitos aleatórios.

Valores de p < 0,05 foram considerados significativos para todas as análises.

## Análise de subgrupos

Os estudos foram divididos em subgrupos para análise de sensibilidade. A análise de subgrupos foi realizada por tipo de medicamento usado nos estudos selecionados (separando os grupos em HBPM/enoxaparina, HNF, fondaparinux e bivalirudina) e por indicação de tratamento (SCA ou TEV). Também realizamos uma análise de sensibilidade incluindo apenas ensaios clínicos randomizados.

## Considerações éticas

Visto que se trata de uma revisão sistemática, não foi necessário submeter o estudo ao Comitê de Ética em Pesquisa. O protocolo do estudo foi publicado anteriormente na plataforma PROSPERO em 30 de abril de 2021 (CRD42021243189). O protocolo do estudo está disponível em https://www.crd.york.ac.uk/prospero/display_record.php?RecordID=243189.

## Resultados

### Identificação e seleção dos estudos

**Figura 1:**
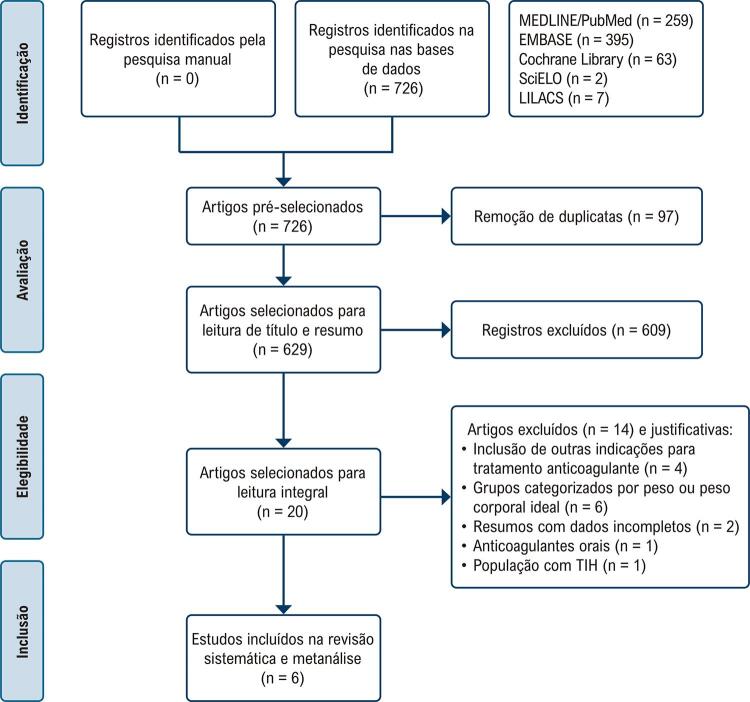
– Fluxograma PRISMA (seleção dos artigos). TIH: trombocitopenia induzida por heparina.

A busca nas bases de dados identificou 726 registros, dos quais 97 eram duplicados, restando 629. Nenhum estudo elegível foi encontrado por meio da busca manual. Após o processo de triagem com título e resumo, 20 estudos foram selecionados para leitura completa, dos quais 14 foram excluídos com base nos critérios de elegibilidade. Ao final do processo de seleção, foram incluídos 6 estudos para análise qualitativa, bem como análise quantitativa (metanálise), conforme mostrado na Figura 1.

### Características dos estudos incluídos

Os estudos excluídos com seus respectivos critérios de exclusão são apresentados na Figura 1. No final, foram selecionados 6 estudos para a revisão sistemática, dos quais 3 eram coortes retrospectivas e 3 eram análises post-hoc de ensaios clínicos randomizados (Tabela 1). Spinler et al.^9^ realizaram uma análise de subgrupo com base nos dados combinados dos ensaios clínicos ESSENCE e TIMI 11B, enquanto Mahaffey et al.^10^ usaram a base de dados do ensaio SYNERGY. Os 3 estudos randomizados compararam enoxaparina a HNF para o tratamento de SCA. Davidson et al.^11^ usaram dados dos ensaios clínicos MATISSE, que testaram fondaparinux para o tratamento de TEV como uma alternativa ao tratamento clássico com heparina (HNF, enoxaparina). Os detalhes das estratégias anticoagulantes adotadas em cada estudo estão disponíveis nos Resultados Suplementares.

**Tabela 1:** – Características gerais dos estudos selecionados e suas populações

Estudos	País	Desenho do estudo	Duração total do estudo	Indicação de anticoagulante	Anticoagulante usado	Critérios de elegibilidade	Definição de exposição	Tamanho da amostra	Obesidade, n (%)	Sexo masculino, n (%)
*Spinler 2003*^9^	Estados Unidos	Análise post-hoc de ECR (ESSENCE e TIMI 11B)	ESSENCE: outubro de 1994 a maio de 1996 TIMI 11B: agosto de 1996 a março 1998	SCA	ESSENCE: enoxaparina ou HNF TIMI 11B: enoxaparina ou HNF	Pacientes com SCASST que receberam HNF ou enoxaparina intravenosa contínua. Aqueles com CrCl < 30 mL/min (ESSENCE) ou creatinina sérica ≥ 2,0 mg/dL (TIMI 11B) foram excluídos dos ensaios.	A obesidade foi definida como IMC ≥ 30 kg/m^2^.	6997	1839 (26,3%)	4593 (65,6%)
*Davidson 2007*^11^	Estados Unidos	Análise post-hoc de ECR (MATISSE-DVT e MATISSE-PE)	MATISSE-DVT: abril de 2000 a julho 2001 MATISSE-PE: maio de 2000 a março de 2002	TEV	MATISSE-DVT: fondaparinux ou enoxaparina MATISSE-PE: fondaparinux ou HNF	Foram incluídos dados para pacientes que receberam pelo menos 1 dose do medicamento do estudo (para o tratamento de TEV) e que tinham resultados disponíveis para os desfechos primários do estudo (recorrência de TEV ou sangramento).	A obesidade foi definida como IMC ≥ 30 kg/m^2^.	4327	1216 (28,1%)	2091 (48,3%)
*Spinler 2009*^5^	Estados Unidos	Estudo de coorte retrospectivo observacional	Janeiro de 2004 a março 2006	SCA	Enoxaparina	Pacientes inscritos no CRUSADE entre 1º de janeiro de 2004 e 31 de março de 2006, que receberam enoxaparina para o tratamento inicial de SCASST cujas informações sobre a dose inicial de enoxaparina foram coletadas. Foram excluídos pacientes que foram transferidos para outra instituição, não tinham IMC documentado, não tinham CrCl estimado documentado ou tinham CrCl < 30 ml/min, ou foram submetidos a cirurgia de revascularização do miocárdio durante a hospitalização.	Os pacientes foram divididos em 4 grupos com base no IMC: < 18,5; 18,5 a 24,9; 25,0 a 29,9; ≥ 30 kg/m^2^.	19.061	7428 (39%)	11613 (60,9%)
*Mahaffey 2010*^10^	Estados Unidos	Análise post-hoc de ECR (SYNERGY)	SYNERGY: agosto de 2001 a dezembro de 2003	SCA	SYNERGY: enoxaparina ou HNF	Pacientes com SCASST. Pacientes foram excluídos quando apresentavam contraindicações para HNF ou enoxaparina, foram submetidos a ICP ou terapia trombolítica nas últimas 24 horas, estavam em alto risco de complicações hemorrágicas devido a acidente vascular cerebral ou cirurgia recente, tinham INR > 1,5, tinham distúrbio hemorrágico passado ou presente, ou apresentavam CrCl < 30 mL/min.	Os pacientes foram divididos em 5 grupos com base no IMC: < 20; 20 a 25; 25 a 30; 30 a 35; ≥ 35 kg/m^2^.	9837	3137 (31,9%)	6514 (66,2%)
*Hosch 2017*^12^	Estados Unidos	Estudo de coorte retrospectivo observacional	Julho de 2013 a julho de 2015	TEV	HNF	Pacientes ≥ 18 anos que receberam HNF para tratamento de TEV (TVP e/ou EP), manejados pelo protocolo de dosagem de farmácia. Foram excluídos pacientes que obtiveram TTPa terapêutico menos de 6 horas após o início da heparina.	Os pacientes foram divididos em 3 grupos com base no IMC: < 30; 30 a 40; ≥ 40 kg/m^2^.	294	173 (58,8%)	143 (48,6%)
*Shlensky 2020*^13^	Estados Unidos	Estudo de coorte retrospectivo observacional	Janeiro de 2010 a dezembro de 2016	TEV	HNF	Pacientes ≥ 18 anos de idade que documentaram TEV como indicação para heparina e estavam em NHAI por pelo menos 24 horas. Foram excluídos pacientes que não consentiram a revisão dos seus registros médicos para pesquisa, receberam uma trombectomia (devido ao aumento do risco de sangramento) ou receberam um agente fibrinolítico.	Os pacientes foram divididos em 3 grupos com base no IMC: < 30; 30 a 40; ≥ 40 kg/m^2^.	423	193 (45,6%)	223 (52,7%)

ClCr: depuração de creatinina; EP: embolia pulmonar; HNF: heparina não fracionada; ICP: intervenção coronária percutânea; IMC: índice de massa corporal; NHAI: nomograma de heparina de alta intensidade; RNI: razão normalizada internacional; SCA: síndrome coronária aguda; SCASST: síndrome coronária aguda sem supradesnivelamento do segmento ST; TEV: tromboembolismo venoso; TTPa: tempo de tromboplastina parcial ativada; TVP: trombose venosa profunda. O nível de significância adotado por todos os estudos foi de 5%.

Foram incluídos na revisão 40.939 pacientes que receberam anticoagulação para o tratamento de SCA ou TEV, entre 1994 e 2016, dos quais 35.895 (87,7%) apresentaram SCA. Em apenas 1 estudo,^12^ o grupo obeso representou mais da metade da população estudada (58,8%), enquanto, no geral, essa prevalência foi de 34,2%. A prevalência do sexo masculino foi de 61,5%. Todos os estudos tiveram os Estados Unidos como país sede; no entanto, os pacientes foram recrutados de diferentes regiões do mundo, como Europa, América do Norte, América do Sul e Oceania. Os critérios de elegibilidade e a definição de exposição de cada estudo incluído podem ser observados na Tabela 1.

### Risco de viés nos estudos incluídos

**Figura 2:**
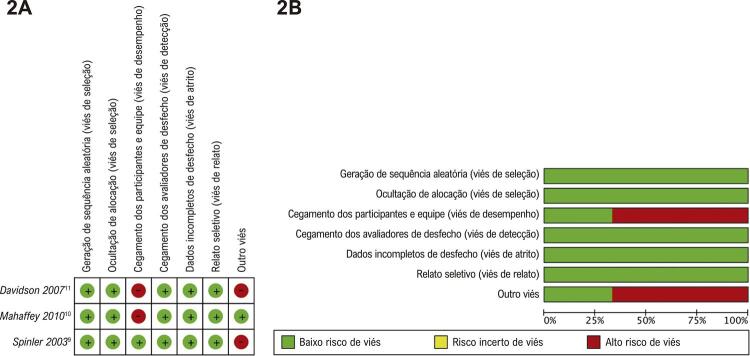
– Análise de risco de viés para ensaios clínicos.

Os estudos caracterizados como análises post-hoc de ensaios clínicos randomizados apresentaram fragilidades metodológicas relacionadas à alocação conhecida e outros vieses, conforme apresentado nas Figuras 2A e 2B. Em Davidson et al.^11^ e Mahaffey et al.,^10^ não houve cegamento dos participantes e avaliadores em relação ao tipo de intervenção aplicada, justificado pelas diferentes vias de administração e dosagem dos medicamentos (HNF por via intravenosa; enoxaparina e fondaparinux por via subcutânea). Spinler et al.^9^ e Davidson et al.,^11^ por outro lado, obtiveram suas análises por meio da fusão de duas bases de dados de ensaios clínicos randomizados, aumentando o risco de viés na amostra selecionada, devido ao comprometimento da randomização e da metodologia adotada.

Em relação aos estudos de coorte, o estudo de Spinler et al.^5^ foi classificado como baixo risco de viés, totalizando 9 estrelas. Foi observado um período de acompanhamento limitado em Shlensky et al.^13^ e Hosch et al.^12^ Este último também apresentou fragilidades metodológicas relacionadas à comparabilidade entre os grupos (Tabela 2).

**Tabela 2:** – Análise de risco de viés para os estudos observacionais

Estudos	Seleção	Comparabilidade	Desfechos
*Spinler 2009*^5^	****	**	***
*Hosch 2017*^12^	****	-	**
*Shlensky 2020*^13^	****	**	**

A análise dos gráficos de funil nas Figuras Suplementares 1A e 1B permite inferir um baixo risco de viés de publicação para todos os artigos incluídos na metanálise, apesar do número reduzido de estudos incluídos.

### Principais achados dos estudos

A Tabela 3 mostra a incidência dos desfechos clínicos avaliados pelos estudos, de acordo com a medicação utilizada, para os grupos obesos e não obesos. Todos os estudos incluídos na revisão avaliaram a taxa de sangramento maior durante a hospitalização, que foi de 6,0% (IC 95%: 5,6% a 6,4%) em pacientes obesos e 6,2% (IC 95%: 5,9% a 6,5%) em pacientes não obesos. As definições de sangramento maior aplicadas em cada estudo são exibidas nos Resultados Suplementares. Em relação aos desfechos de eficácia, apenas 2 estudos analisaram a mortalidade entre 30 e 43 dias de acompanhamento. A taxa de mortalidade no grupo obeso foi de 2,6% (IC 95%: 2,2% a 3,0%), e no grupo não obeso foi de 3,6% (IC 95%: 3,3% a 4,0%). Apenas Spinler et al.^9^ relataram infarto do miocárdio recorrente, enquanto o desfecho de acidente vascular cerebral não foi medido em nenhum dos estudos.

**Tabela 3:** – Desfechos clínicos para pacientes obesos versus não obesos por tipo de antitrombótico usado

Estudos	Anticoagulante (indicação)	Desfecho	Acompanhamento	Incidência em obesos n/N (%)	Incidência em não obesos n/N (%)
*Spinler 2003*^9^	Enoxaparina (SCA)	Óbito	43 dias	24/921 (2,6%)	91/2595 (3,5%)
		IAM	43 dias	45/921 (4,9%)	125/2595 (4,8%)
		RU	43 dias	83/921 (9%)	257/2595 (9,9%)
		Óbito/IAM/RU	43 dias	132/921 (14,3%)	418/2595 (16,1%)
		Sangramento maior	Hospitalização	4/921 (0,4%)	41/2595 (1,6%)
		Qualquer sangramento	Hospitalização	107/921 (11,7%)	243/2595 (9,5%)
	HNF (SCA)	Óbito	43 dias	23/918 (2,5%)	113/2563 (4,4%)
		IAM	43 dias	56/918 (6,1%)	154/2563 (6%)
		RU	43 dias	107/918 (11,7%)	308/2563 (12%)
		Óbito/IAM/RU	43 dias	165/918 (18,0%)	492/2563 (19,2%)
		Sangramento maior	Hospitalização	11/918 (1,2%)	25/2563 (1%)
		Qualquer sangramento	Hospitalização	48/918 (5,3%)	101/2563 (4%)
*Davidson 2007*^11^	Fondaparinux (TEV)	Recorrência de TEV	3 meses	22/594 (3,7%)	61/1560 (3,9%)
		Sangramento maior	Hospitalização	2/590 (0,3%)	23/1549 (1,5%)
	Heparinas* (TEV)	Recorrência de TEV	3 meses	30/622 (4,8%)	70/1551 (4,5%)
		Sangramento maior	Hospitalização	7/611 (1,1%)	18/1540 (1,2%)
*Spinler 2009*^5^	Enoxaparina (SCA)	Sangramento maior	Hospitalização	513/7428 (6,9%)	900/11,633 (7,7%)
*Mahaffey 2010*^10^	Enoxaparina (SCA)	Óbito/IAM	30 dias	202/1585 (12,8%)	486/3331 (14,5%)
			6 meses	262/1585 (16,5%)	605/3331 (18,2%)
		Óbito	30 dias	37/1585 (2,3%)	119/3331 (3,6%)
			6 meses	69/1585 (4,4%)	209/3331 (6,3%)
			1 ano	108/1585 (6,8%)	269/3331 (8%)
		Sangramento grave (GUSTO)	Hospitalização	39/1585 (2,5%)	94/3331 (2,8%)
		Sangramento maior (TIMI)	Hospitalização	138/1585 (8,7%)	308/3331 (9,2%)
	HNF (SCA)	Óbito/IAM	30 dias	206/1552 (13,3%)	515/3369 (15,3%)
			6 meses	253/1552 (16,3%)	628/3369 (18,6%)
		Óbito	30 dias	45/1552 (2,9%)	110/3369 (3,3%)
			6 meses	75/1552 (4,8%)	182/3369 (5,4%)
			1 ano	95/1552 (6,1%)	262/3369 (7,8%)
		Sangramento grave (GUSTO)	Hospitalização	28/1552 (1,8%)	76/3369 (2,3%)
		Sangramento maior (TIMI)	Hospitalização	111/1552 (7,2%)	257/3369 (7,6%)
*Hosch 2017*^12^	HNF (TEV)	Evento hemorrágico Queda de Hb ≥ 2 g/dL e ≥ 2 unidades de CH recebidas	Hospitalização	20/173 (11,5%)	17/121 (14%)
			Hospitalização	3/173 (1,7%)	4/121 (3,3%)
		Eventos trombóticos	Hospitalização	0/173	0/121
*Shlensky 2020*^13^	HNF (TEV)	Sangramento maior	Hospitalização	13/193 (6,7%)	14/230 (6,1%)
		Complicações trombóticas	Hospitalização	1/193 (0,5%)	1/230 (0,4%)

CH: concentrado de hemácias; GUSTO: Global Use of Strategies to Open Occluded Arteries; Hb: hemoglobina; HNF: heparina não fracionada; IAM: infarto agudo do miocárdio; RU: revascularização urgente; SCA: síndrome coronária aguda; TIMI: Thrombolysis in Myocardial Infarction; VTE: tromboembolismo venoso. *UFH em MATISSE-EP e enoxaparina em MATISSE-TVP.

### Resumo dos resultados

#### Sangramento maior

**Figura 3:**
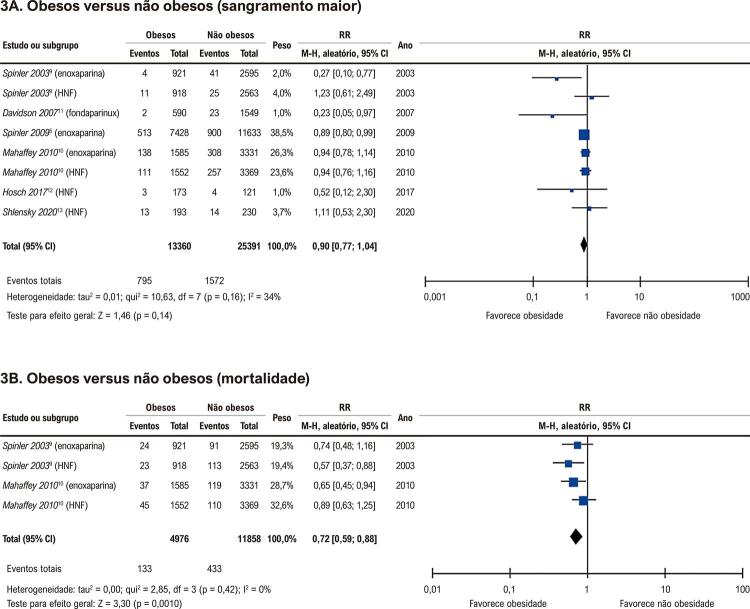
– Gráficos de floresta. HNF: heparina não fracionada; IC: intervalo de confiança; M-H: análise de Mantel-Haenszel; RR: risk ratio.

Os resultados da presente análise mostraram que a taxa de sangramento maior durante o período intra-hospitalar foi semelhante entre pacientes obesos e não obesos usando anticoagulantes parenterais, conforme mostrado na Figura 3A. A heterogeneidade foi considerada moderada. A incidência do desfecho permaneceu comparável quando os estudos sobre SCA e TEV foram analisados separadamente, bem como quando analisados por anticoagulante (enoxaparina ou HNF). A taxa de sangramento também foi semelhante entre os grupos quando os ensaios clínicos randomizados foram analisados separadamente. Os gráficos de floresta das análises de subgrupos são apresentados nas Figuras Suplementares 2 a 6.

#### Mortalidade

Apenas 2 artigos avaliaram a incidência de morte, ambos no cenário de SCA.^9,10^ Durante um período de acompanhamento de 30 a 43 dias, a taxa de mortalidade foi menor em pacientes obesos em comparação com pacientes não obesos, como mostrado na Figura 3B. A heterogeneidade foi considerada baixa. Não foi possível realizar uma análise individualizada para cada agente antitrombótico devido ao pequeno número de artigos na análise.

#### Outros desfechos

Para os outros desfechos previamente estabelecidos (infarto do miocárdio e acidente vascular cerebral), a metanálise não foi realizada devido ao número limitado de artigos. Apenas 1 estudo (Spinler et al.^9^) avaliou o desfecho do infarto do miocárdio isoladamente, enquanto a incidência de acidente vascular cerebral não foi medida em nenhum dos estudos incluídos na revisão.

#### Qualidade da evidência (GRADE)

A Tabela Suplementar 2 fornece um resumo da avaliação da qualidade da evidência usando a abordagem GRADE. Para o desfecho de sangramento maior, a evidência foi inicialmente classificada como moderada, pois incluiu estudos randomizados e observacionais. Após avaliar cada domínio, julgamos a qualidade da evidência como baixa, com um rebaixamento de 1 ponto no domínio de risco de viés. Para o desfecho de mortalidade, a evidência foi inicialmente classificada como alta, pois foi derivada exclusivamente de ensaios clínicos randomizados. Após um rebaixamento de 1 ponto no domínio de risco de viés, foi classificada como qualidade moderada. Nenhuma característica foi identificada para aumentar o nível de evidência. A avaliação detalhada através do método GRADE pode ser encontrada nas Tabelas Suplementares 3 e 4.

## Discussão

O presente estudo avaliou a influência da obesidade na segurança e eficácia do tratamento antitrombótico após eventos coronários agudos ou TEV. Verificamos que a obesidade tem pouco ou nenhum impacto na incidência de desfechos clínicos após terapia anticoagulante. Poucos estudos relataram desfechos trombóticos e de óbito, limitando as conclusões sobre a eficácia. Por outro lado, em relação aos eventos hemorrágicos, o uso de HNF, enoxaparina e fondaparinux parece ser seguro na população obesa (IMC ≥ 30 kg/m^2^).

### Síndrome coronariana aguda

No contexto da SCA, na análise combinada dos ensaios ESSENCE e TIMI 11B, não houve diferença entre pacientes obesos (IMC ≥ 30 kg/m^2^) e não obesos na incidência do desfecho primário composto de óbito, infarto do miocárdio e necessidade de revascularização urgente ou ocorrência de sangramento importante. No entanto, houve uma tendência para um risco maior de qualquer sangramento entre aqueles com IMC mais alto.^9^ Ademais, a incidência de sangramento maior foi semelhante entre pacientes obesos e não obesos tratados com HNF, mas favoreceu pacientes com IMC ≥ 30 kg/m^2^ entre aqueles tratados com enoxaparina (RR: 0,27, IC 95%: 0,10 a 0,77) (Figura 3A). Dados do ensaio SYNERGY, no qual 9.873 pacientes foram randomizados para receber HNF ou enoxaparina, não verificaram uma diferença significativa na ocorrência de óbito, infarto do miocárdio e sangramento maior associado ao IMC.^10^ Esses achados, confirmados na presente metanálise, apoiam a abordagem baseada no peso sem um limite de dose (Figura 3A).

Por outro lado, Spinler et al.,^5^ com base no registro CRUSADE, mostraram que pacientes com sobrepeso e obesidade tendem a ser tratados com doses reduzidas de enoxaparina (< 0,95 mg/kg), quando comparados àqueles com peso normal. Além disso, observou-se que pacientes com peso > 150 kg nessa coorte, embora sub-representados (n = 37), que foram tratados com doses recomendadas de enoxaparina (0,95 a 1,05 mg/kg), apresentaram maior risco de sangramento quando comparados àqueles que receberam uma dose reduzida. Isso pode explicar por que a presente metanálise não foi capaz de demonstrar diferença nas taxas de sangramento entre pacientes obesos e não obesos. No entanto, o pequeno tamanho da amostra e o caráter observacional do estudo impedem conclusões definitivas.

O estudo OASIS-5 mostrou que o fondaparinux foi não inferior à enoxaparina quanto à ocorrência de eventos isquêmicos após NSTEMI ou angina instável, e foi associado a uma redução significativa no risco de sangramento maior e mortalidade em 30 e 180 dias. No entanto, houve uma baixa representatividade da população obesa no estudo.^6^ Assim, o questionamento acerca da dose fixa de 2,5 mg em pacientes obesos ainda é incerta. Em uma análise interna, entre os pacientes que usaram fondaparinux, a incidência de sangramento durante o tratamento e no dia 9 foi inversamente proporcional ao peso corporal, e aqueles com peso > 100 kg tiveram melhor prognóstico,^14^ sugerindo que a segurança desse tratamento pode não ser afetada pela presença da obesidade. Não foram encontrados estudos que comparassem a incidência de desfechos clínicos entre pacientes obesos e não obesos em uso de fondaparinux.

Em relação ao desfecho de mortalidade, a síntese dos resultados dos estudos de Spinler et al.^9^ e Mahaffey et al.^10^ mostrou uma tendência de melhor prognóstico entre os pacientes obesos no período de 30 a 43 dias, achado previamente descrito na literatura^15-17^ como o “paradoxo da obesidade”. O fato de os pacientes obesos serem frequentemente mais jovens, menos frágeis e apresentarem menos preditores de risco para sangramento no momento da internação hospitalar pode justificar resultados relacionados a uma menor ocorrência de desfechos clínicos nesse grupo populacional. Entretanto, a incapacidade de realizar ajustes estatísticos, característica do nosso desenho de estudo, dificulta a exclusão da possibilidade de que variáveis de confusão e estratégias de tratamento diferenciadas na população obesa possam ser responsáveis pela diferente taxa de mortalidade ou mesmo por não encontrar diferença quanto aos eventos hemorrágicos.

### Tromboembolismo venoso

Barletta et al. compararam os valores do tempo de tromboplastina parcial ativada (TTPa) alcançados por pacientes com e sem obesidade mórbida (IMC > 40 kg/m^2^), usando o nomograma baseado no peso para o tratamento de TEV. Os autores mostraram que aqueles pertencentes ao grupo de maior peso tiveram uma maior incidência de overdose e que o IMC foi um preditor independente de valores de TTPa supraterapêuticos, sugerindo uma possível necessidade de um limite de dose para aqueles com IMC > 40 kg/m^2^.^18^

Nesse cenário, 2 estudos nesta revisão visaram estudar a influência do IMC na eficácia e segurança da HNF em pacientes com TEV. No estudo de Hosch et al.,^12^ os pacientes recebiam a dose de HNF com base no peso corporal, exceto quando seu peso excedia 20% do peso corporal ideal. Nestes casos, a dose foi baseada no peso corporal ideal. Em contraste, Shlensky et al.^13^ conduziram o estudo no cenário em que todos os pacientes, independentemente do peso, receberam a mesma dosagem de medicamento com base no peso corporal. Apesar das diferentes abordagens, nenhum estudo encontrou diferença entre as 3 classes de IMC (< 30, 30 a 40 e > 40 kg/m^2^) em relação ao tempo necessário para atingir o primeiro valor terapêutico de TTPa e a ocorrência de sangramento durante o estudo. Corroborando os achados de Barletta et al.,^18^ Shlensky et al.^13^ também identificaram maior incidência de valores supraterapêuticos de TTPa no grupo de pacientes com obesidade mórbida; no entanto, esse achado não se refletiu na ocorrência de sangramento, sugerindo que não há impacto clínico do IMC na segurança da HNF em TEV. Além do pequeno tamanho da amostra, os estudos têm a limitação de identificar eventos de sangramento por meio do resumo da alta hospitalar, o que pode potencialmente subestimar o número de eventos.

O fondaparinux foi testado como uma opção alternativa às heparinas (HNF e enoxaparina) nos ensaios clínicos MATISSE, que demonstraram a não inferioridade deste medicamento quando utilizado em 3 doses baseadas no peso (5,0, 7,5 e 10 mg).^19,20^ Davidson et al.,^11^ com base nos dados do MATISSE, mostraram que os resultados foram mantidos no subgrupo de pacientes obesos (IMC > 30), corroborando os achados do presente estudo. Em nossa metanálise, apesar do amplo intervalo de confiança, os resultados mostraram que pacientes obesos em uso de fondaparinux tenderam a ter uma taxa reduzida de sangramento maior em comparação aos pacientes não obesos (RR: 0,23, IC 95%: 0,05 a 0,97) (Figura 3A). No estudo MATISSE, embora tenha havido um ajuste de dose com base no peso na administração de fondaparinux (5 mg para < 50 kg; 7,5 mg para 50 a 100 kg; 10 mg para > 100 kg), todos os pacientes com peso > 100 kg receberam a mesma dose diária de 10 mg, sem progressão da dose baseada no peso, o que pode ser um potencial fator de proteção contra sangramento para pacientes com obesidade mórbida.

### Limitações

A presente revisão tem algumas limitações. Devido à alta prevalência de pacientes com SCA (87,7%) na amostra estudada, esta análise diz respeito principalmente a pacientes que sofreram um evento coronário agudo, com conclusões limitadas sobre pacientes com TEV. A classificação dos eventos hemorrágicos e as estratégias de anticoagulação adotadas foram diversas, limitando a combinação desses dados. Devido às variações na definição e na coleta de desfechos, não foi possível realizar uma análise dos principais desfechos combinados. Ademais, devido à incapacidade de controlar variáveis de confusão, outros fatores como o uso de outros antiplaquetários e anticoagulantes e as diferenças no perfil clínico entre pacientes obesos e não obesos (idade, sexo, comorbidades) podem ter influenciado os resultados obtidos. A população também variou entre os estudos. Estudos futuros com análise de meta-regressão, que possam controlar essas variáveis, seriam de interesse. Além disso, o longo período de inclusão dos estudos (2003 a 2020), associado a avanços significativos no tratamento adjuvante da SCA durante esse período, pode ter influenciado os resultados. Por fim, pacientes com obesidade grau III ainda são sub-representados, tornando necessários estudos focados nessa população específica.

### Implicações para a prática e pesquisas futuras

Nossos resultados, embora geradores de hipóteses, sugerem que não é necessário um tratamento diferenciado para pacientes obesos com SCA ou TEV, no que diz respeito a terapia anticoagulante. Futuros estudos randomizados na população específica de pacientes obesos são necessários para obter conclusões mais robustas.

## Conclusões

Nos pacientes tratados para TEV ou SCA, as taxas de sangramento foram comparáveis entre pacientes obesos e não obesos, independentemente do anticoagulante usado. A menor taxa de mortalidade observada nos pacientes obesos pode representar o efeito de fatores de confusão não contabilizados. Estudos subsequentes são necessários para validar estes achados.

## S-1 *Material suplementar

Para informação adicional, por favor, clique aqui http://abccardiol.org/supplementary-material/2025/12204/2024-0544_AO_Supplemental_Material_obesity_and_antithrombotics.pdf
