# Supplementary material for: Influência da Obesidade na Segurança e Eficácia do Tratamento Antitrombótico: Uma Revisão Sistemática e Metanálise
Source: Arq Bras Cardiol. 2025 May 12;122(4):e20240544. [Article in Portuguese] doi: 10.36660/abc.20240544 (PMC12108139; doi:10.36660/abc.20240544)
Supplement: Supplementary file 1 [file 0066-782x-abc-122-4-e20240544-suppl01-en.pdf]

SUPPLEMENTAL MATERIAL

SUPPLEMENTAL METHODS

Databases and Boolean strategies used

Data base	Search strategy	Results
Pubmed	(("Acute Coronary Syndrome"[Mesh] OR "Acute Coronary Syndromes" OR "Coronary Syndrome, Acute" OR "Coronary Syndromes, Acute" OR "Syndrome, Acute Coronary" OR "Syndromes, Acute Coronary" OR "Angina, Unstable"[Mesh] OR "Anginas, Unstable" OR "Unstable Anginas" OR "Angina Pectoris, Unstable" OR "Angina Pectori, Unstable" OR "Unstable Angina Pectori" OR "Unstable Angina Pectoris" OR "Unstable Angina" OR "Angina at Rest" OR "Angina, Preinfarction" OR "Anginas, Preinfarction" OR "Preinfarction Angina" OR "Preinfarction Anginas" OR "Myocardial Preinfarction Syndrome" OR "Myocardial Preinfarction Syndromes" OR "Preinfarction Syndrome, Myocardial" OR "Preinfarction Syndromes, Myocardial" OR "Syndrome, Myocardial Preinfarction" OR "Syndromes, Myocardial Preinfarction" OR "Non-ST Elevated Myocardial Infarction"[Mesh] OR "Non ST Elevated Myocardial Infarction" OR "NSTEMI" OR "Non-ST-Elevation Myocardial Infarction" OR "Infarction, Non-ST-Elevation Myocardial" OR "Infarctions, Non-ST-Elevation Myocardial" OR "Myocardial Infarction, Non-ST-Elevation" OR "Myocardial Infarctions, Non-ST-Elevation" OR "Non ST Elevation Myocardial Infarction" OR "Non-ST-Elevation Myocardial Infarctions" OR "Myocardial Infarction"[Mesh] OR "Infarction, Myocardial" OR "Infarctions, Myocardial" OR "Myocardial Infarctions" OR "Cardiovascular Stroke" OR "Cardiovascular Strokes" OR "Stroke, Cardiovascular" OR "Strokes, Cardiovascular" OR "Myocardial Infarct" OR "Infarct, Myocardial" OR "Infarcts, Myocardial" OR "Myocardial Infarcts" OR "Heart Attack" OR "Heart Attacks" OR "ST Elevation Myocardial Infarction"[Mesh] OR "ST Segment Elevation Myocardial Infarction" OR "ST Elevated Myocardial Infarction" OR "STEMI" OR "Anterior Wall Myocardial Infarction"[Mesh] OR "Myocardial Infarction, Anterior Wall" OR "Anterolateral Myocardial Infarction" OR "Anterolateral Myocardial Infarctions" OR "Infarction, Anterolateral Myocardial" OR "Infarctions, Anterolateral Myocardial" OR "Myocardial Infarction, Anterolateral" OR "Myocardial Infarctions, Anterolateral" OR "Anteroseptal Myocardial Infarction" OR "Anteroseptal Myocardial Infarctions" OR "Infarction, Anteroseptal Myocardial" OR "Infarctions, Anteroseptal Myocardial" OR "Myocardial Infarction, Anteroseptal" OR "Myocardial Infarctions, Anteroseptal" OR "Acute Anterior Wall Myocardial Infarction" OR "Inferior Wall Myocardial Infarction"[Mesh] OR "Diaphragmatic Myocardial Infarction" OR "Diaphragmatic Myocardial Infarctions" OR "Infarction, Diaphragmatic Myocardial" OR "Infarctions, Diaphragmatic Myocardial" OR "Myocardial Infarction, Diaphragmatic" OR "Myocardial Infarctions, Diaphragmatic" OR "Myocardial Infarction, Inferior Wall" OR "Inferior Myocardial Infarction" OR "Infarction, Inferior Myocardial" OR "Infarctions, Inferior Myocardial" OR "Inferior Myocardial Infarctions" OR "Myocardial Infarction, Inferior" OR "Myocardial Infarctions, Inferior" OR "Acute Inferior Myocardial Infarction" OR "Venous Thromboembolism"[Mesh] OR "Thromboembolism, Venous" OR "Venous Thrombosis"[Mesh] OR "Phlebothrombosis" OR "Phlebothromboses" OR "Thrombosis, Venous" OR "Thromboses, Venous" OR "Venous Thromboses" OR "Deep Vein Thrombosis" OR "Deep Vein Thromboses" OR "Thromboses, Deep Vein" OR "Vein Thromboses, Deep" OR "Vein Thrombosis, Deep" OR "Deep-Venous Thrombosis" OR "Deep-Venous Thromboses" OR "Thromboses, Deep-Venous" OR "Thrombosis, Deep-Venous" OR "Deep-Vein Thrombosis" OR "Deep-Vein Thromboses" OR "Thromboses, Deep-Vein" OR "Thrombosis, Deep-Vein" OR "Thrombosis, Deep Vein" OR "Deep Venous Thrombosis" OR "Deep Venous Thromboses" OR "Thromboses, Deep Venous" OR "Thrombosis, Deep Venous" OR "Venous Thromboses, Deep" OR "Venous Thrombosis, Deep" OR "Pulmonary Embolism"[Mesh] OR "Pulmonary Embolisms" OR "Embolism, Pulmonary" OR "Embolisms, Pulmonary" OR "Pulmonary	249

	Thromboembolisms" OR "Pulmonary Thromboembolism" OR "Thromboembolism, Pulmonary" OR "Thromboembolisms, Pulmonary") AND ("Heparin"[Mesh] OR "Unfractionated Heparin" OR "Heparin, Unfractionated" OR "Heparinic Acid" OR "Liquaemin" OR "Sodium Heparin" OR "Heparin, Sodium" OR "Heparin Sodium" OR "alpha-Heparin" OR "alpha Heparin" OR "Heparin, Low-Molecular-Weight"[Mesh] OR "Heparin, Low Molecular Weight" OR "LMWH" OR "Low Molecular Weight Heparin" OR "Low-Molecular-Weight Heparin" OR "Enoxaparin"[Mesh] OR "Enoxaparine" OR "PK-10,169" OR "PK 10,169" OR "PK10,169" OR "PK-10169" OR "PK 10169" OR "PK10169" OR "EMT-967" OR "EMT 967" OR "EMT967" OR "Lovenox" OR "Clexane" OR "EMT-966" OR "EMT 966" OR "EMT966" OR "Nadroparin"[Mesh] OR "Nadroparine" OR "Nadroparin Calcium" OR "Calcium, Nadroparin" OR "Fraxiparin" OR "Fraxiparine" OR "CY 216" OR "CY-216" OR "CY216" OR "LMF CY-216" OR "LMF CY 216" OR "LMF CY216" OR "Dalteparin"[Mesh] OR "Tedelparin" OR "FR-860" OR "FR 860" OR "FR860" OR "Kabi-2165" OR "Kabi 2165" OR "Kabi2165" OR "Dalteparin Sodium" OR "Sodium, Dalteparin" OR "Fragmin" OR "Fragmine" OR "Tinzaparin"[Mesh] OR "2-Propenoic acid, 3-phenyl-" OR "2 Propenoic acid, 3 phenyl" OR "3-phenyl- 2-Propenoic acid" OR "Tinzaparin Sodium" OR "Innohep" OR "Fondaparinux"[Mesh] OR "Fondaparinux Sodium" OR "Quixidar" OR "Arixtra" OR "bivalirudin" [Supplementary Concept] OR "Phe-Pro-Arg-Pro-(Gly)4-desulfohirudin-(53-64)" OR "Phe-Pro-Arg-Pro-(Gly)4 desulfato-Tyr63'-hirugen" OR "Phe-Pro-Arg-Pro-(Gly)4-Asn-Gly-Asp-Phe-Glu-Glu-Ile-Pro-Glu-Glu-Tyr-Leu" OR "CTB-001" OR "Hirulog" OR "Hirulog-1" OR "Angiomax" OR "BG 8967" OR "BG8967" OR "BG-8967")) AND (("Obesity"[Mesh]) OR "Obesity, Morbid"[Mesh] OR "Overweight"[Mesh] OR "Morbid Obesities" OR "Obesities, Morbid" OR "Obesity, Severe" OR "Obesities, Severe" OR "Severe Obesities" OR "Severe Obesity" OR "Morbid Obesity")	
EMBASE	('acute coronary syndrome'/exp OR 'acute coronary syndrome' OR 'acute coronary syndromes' OR 'heart infarction'/exp OR 'cardiac infarct' OR 'cardiac infarction' OR 'cardial infarct' OR 'heart attack' OR 'heart infarct' OR 'heart infarction' OR 'heart micro infarction' OR 'heart muscle infarction' OR 'infarction, heart' OR 'myocardial infarct' OR 'myocardial infarction' OR 'myocardium infarct' OR 'myocardium infarction' OR 'premonitory infarction sign' OR 'second heart attack' OR 'subendocardial infarction' OR 'transmural cardiac infarction' OR 'transmural heart infarction' OR 'transmural infarction, heart' OR 'unstable angina pectoris'/exp OR 'angina pectoris, unstable' OR 'angina, unstable' OR 'unstable angina' OR 'unstable angina pectoris' OR 'venous thromboembolism'/exp OR 'thromboembolism, venous' OR 'vein thromboembolism' OR 'venous thromboembolism' OR 'vein thrombosis'/exp OR 'phlebo-thrombosis' OR 'phlebothrombosis' OR 'thrombosis, venous' OR 'vein thrombosis' OR 'vena thrombosis' OR 'venothrombosis' OR 'venothrombotic event' OR 'venous thrombosis' OR 'lung embolism'/exp OR 'chronic lung embolism' OR 'embolism, lung' OR 'lung embolism' OR 'lung embolization' OR 'lung embolus' OR 'lung embolus recurrence' OR 'lung emboly' OR 'lung microembolism' OR 'lung microembolization' OR 'lung microembolus' OR 'lung thromboembolism' OR 'microembolus, lung' OR 'pulmonary embolism' OR 'pulmonary embolization' OR 'pulmonary embolus' OR 'pulmonary microembolism' OR 'pulmonary thromboembolic disease' OR 'pulmonary thromboembolism' OR 'thromboembolism, lung') AND ('heparin'/exp OR 'alpha heparin' OR 'ammonium heparinate' OR 'benzalkonium heparin' OR 'beparine' OR 'clarin' OR 'contusol' OR 'disebrin' OR 'eleparon' OR 'elheparin' OR 'elheparon' OR 'endogenous heparin' OR 'epiheparin' OR 'gag 98' OR 'helberina' OR 'hep flush kit' OR 'hep lock' OR 'hep-lock' OR 'hep-lock u/p' OR 'hep-pak cvc' OR 'hepaflex' OR 'hepalean' OR 'heparin' OR 'heparin injection b.p.' OR 'heparin leo' OR 'heparin lock flush' OR 'heparin lock flush plus sodium chloride' OR 'heparin lock flush preservative free' OR 'heparin monosulfate' OR 'heparin monosulphate' OR 'heparin novo' OR 'heparin ointment' OR 'heparin potassium' OR 'heparin sodium' OR 'heparin sodium 1, 000 units and sodium chloride 0.9%' OR 'heparin sodium 1, 000 units in dextrose 5%' OR 'heparin sodium 1, 000 units in sodium chloride 0.9%' OR 'heparin sodium 10, 000 units in dextrose 5%' OR 'heparin sodium 10, 000 units in sodium chloride 0.45%' OR 'heparin sodium 10, 000 units in sodium chloride 0.9%' OR 'heparin sodium	395

12, 500 units in dextrose 5%' OR 'heparin sodium 12, 500 units in sodium chloride 0.45%' OR 'heparin sodium 12, 500 units in sodium chloride 0.9%' OR 'heparin sodium 2, 000 units and sodium chloride 0.9%' OR 'heparin sodium 2, 000 units in dextrose 5%' OR 'heparin sodium 2, 000 units in sodium chloride 0.9%' OR 'heparin sodium 20, 000 units and dextrose 5%' OR 'heparin sodium 20, 000 units in dextrose 5%' OR 'heparin sodium 25, 000 units and dextrose 5%' OR 'heparin sodium 25, 000 units in dextrose 5%' OR 'heparin sodium 25, 000 units in sodium chloride 0.45%' OR 'heparin sodium 25, 000 units in sodium chloride 0.9%' OR 'heparin sodium 5, 000 units and sodium chloride 0.9%' OR 'heparin sodium 5, 000 units in dextrose 5%' OR 'heparin sodium 5, 000 units in sodium chloride 0.45%' OR 'heparin sodium 5, 000 units in sodium chloride 0.9%' OR 'heparin sodium b braun' OR 'heparin sodium preservative free' OR 'heparin subcutaneous' OR 'heparin sulfate' OR 'heparin sulfuric acid' OR 'heparin sulphate' OR 'heparina' OR 'heparina leo' OR 'heparinate sodium' OR 'heparine' OR 'heparine choay' OR 'heparine novo' OR 'heparinic acid' OR 'heparitin monosulfate' OR 'heparitin monosulphate' OR 'hepcon' OR 'hepflush-10' OR 'hepsal' OR 'inhepar' OR 'inviclot' OR 'lipo hepin' OR 'lipo-hepin' OR 'lipohepin' OR 'liquaemin' OR 'liquaemin lock flush' OR 'liquaemin sodium' OR 'liquaemin sodium preservative free' OR 'liquemin' OR 'liquemine' OR 'menaven' OR 'monoparin' OR 'mucoitin polysulfate' OR 'mucoitin polysulfate ester' OR 'mucoitin polysulphate' OR 'mucoitin polysulphate ester' OR 'mucoitin sodium polysulfate' OR 'mucoitin sodium polysulphate' OR 'multiparin' OR 'nevparin' OR 'noparin' OR 'panheparin' OR 'panhepin' OR 'panheprin' OR 'parinix' OR 'praecivenin' OR 'pularin' OR 'sodium heparin' OR 'thrombareduct' OR 'thrombo vetren' OR 'thromboliquin' OR 'thromboliquine' OR 'thrombophlogat' OR 'thrombophob' OR 'thrombophob gel' OR 'thromboreduct' OR 'thrombosamine' OR 'thrombosamine heparin' OR 'thrombosamine heparine' OR 'unfractionated heparin' OR 'uniparin' OR 'vetren' OR 'vister' OR 'low molecular weight heparin'/exp OR 'bm 2123' OR 'bm2123' OR 'choay' OR 'depolymerized heparin' OR 'ebpm 1' OR 'ebpm 2' OR 'ebpm 3' OR 'ebpm1' OR 'ebpm2' OR 'ebpm3' OR 'ff 1034' OR 'ff1034' OR 'fr 860' OR 'fr860' OR 'gag 869' OR 'heparin lmw 2133' OR 'heparin, low molecular weight' OR 'heparin, low molecular weight fraction' OR 'heparin, low-molecular-weight' OR 'low molecular heparin' OR 'low molecular weight heparin' OR 'nm heparin' OR 'pk 007' OR 'sandoz 5100' OR 'sandoz 6700' OR 'traxyparine' OR 'fondaparinux'/exp OR 'arixtra' OR 'fondaparin' OR 'fondaparin sodium' OR 'fondaparinux' OR 'fondaparinux sodium' OR 'ic 851589' OR 'ic851589' OR 'org 31540' OR 'org31540' OR 'quixidar' OR 'sr 90107' OR 'sr 90107a' OR 'sr90107' OR 'sr90107a' OR 'enoxaparin'/exp OR 'clexan' OR 'clexane' OR 'clexane 40' OR 'clexane forte' OR 'clexane multidose' OR 'clexane t' OR 'decipar' OR 'enoxaparin' OR 'enoxaparin sodium' OR 'inhixa' OR 'klexane' OR 'ledraxen' OR 'lovenox' OR 'lovenox (preservative free)' OR 'neoparin' OR 'neoparin-nx' OR 'pk 10169' OR 'pk10169' OR 'qualiop klinik' OR 'rp 54563' OR 'rp54563' OR 'thorinane' OR 'dalteparin'/exp OR 'dalteparin' OR 'dalteparin sodium' OR 'fragmin' OR 'fragmin p forte' OR 'fragmine' OR 'k 2165' OR 'k2165' OR 'kabi 2165' OR 'low liquemin' OR 'tinzaparin'/exp OR 'innohep' OR 'lhn1' OR 'logiparin' OR 'tinzaparin' OR 'tinzaparin sodium' OR 'nadroparin'/exp OR 'cy 216' OR 'cy 216d' OR 'cy216' OR 'cy216d' OR 'fraxiparin' OR 'fraxiparin multi' OR 'fraxiparina' OR 'fraxiparina forte' OR 'fraxiparine' OR 'fraxiparine forte' OR 'fraxiparine multi' OR 'fraxodi' OR 'nadroparin' OR 'nadroparin calcium' OR 'nadroparin sodium' OR 'seledie' OR 'seleparina' OR 'seleparine' OR 'tedegliparin' OR 'bivalirudin'/exp OR 'angiomax' OR 'angiomax rtu' OR 'angiox' OR 'bg 8967' OR 'bg8967' OR 'bivalirudin' OR 'bivalirudin in sodium chloride 0.9%' OR 'bivalurudin trifluoroacetate' OR 'd phe pro arg pro (gly) 4 desulfohirudin [53-64]' OR 'd phe pro arg pro gly gly gly gly asn gly asp phe glu glu ile pro glu glu tyr leu' OR 'dextro phenylalanylprolylarginylprolylglycylglycylglycylglycylasparaginylglycyl alpha aspartylphenylalanyl alpha glutamyl alpha glutamylisoleucylprolyl alpha glutamyl alpha glutamyl alpha tyrosyleucine' OR 'dextro phenylalanylprolylarginylprolylglycylglycylglycylglycylasparaginylglycyl alpha aspartylphenylalanyl alpha glutamyl alpha glutamylisoleucylprolyl alpha glutamyl alpha glutamyl alpha tyrosyleucine bis (2, 2, 2 trifluoroacetate)' OR 'hirulog' OR 'hirulog 1') AND ('obesity'/exp OR 'adipose tissue hyperplasia' OR 'adipositas' OR 'adiposity' OR

	'alimentary obesity' OR 'body weight, excess' OR 'corpulency' OR 'fat overload syndrome' OR 'nutritional obesity' OR 'obesitas' OR 'obesity' OR 'overweight' OR 'morbid obesity'/exp OR 'morbid obesity' OR 'obesity, morbid') AND ('cardiovascular disease'/exp OR 'angiocardiopathy' OR 'angiocardiovascular disease' OR 'cardiovascular complication' OR 'cardiovascular disease' OR 'cardiovascular diseases' OR 'cardiovascular disorder' OR 'cardiovascular disturbance' OR 'cardiovascular lesion' OR 'cardiovascular syndrome' OR 'cardiovascular vegetative disorder' OR 'complication, cardiovascular' OR 'disease, cardiovascular' OR 'major adverse cardiovascular event' OR 'bleeding'/exp OR 'abnormal bleeding' OR 'bleeding' OR 'bleeding complication' OR 'blood effusion' OR 'blood loss' OR 'capillary bleeding' OR 'haemorrhage' OR 'haemorrhage model' OR 'haemorrhagic activity' OR 'hemorrhage' OR 'hemorrhage model' OR 'hemorrhagia' OR 'hemorrhagic activity' OR 'spontaneous haemorrhage' OR 'spontaneous hemorrhage') AND ('randomized controlled trial'/exp OR 'controlled trial, randomized' OR 'randomised controlled study' OR 'randomised controlled trial' OR 'randomized controlled study' OR 'randomized controlled trial' OR 'trial, randomized controlled' OR 'observational study'/exp OR 'non experimental studies' OR 'non experimental study' OR 'nonexperimental studies' OR 'nonexperimental study' OR 'observation studies' OR 'observation study' OR 'observational studies' OR 'observational studies as topic' OR 'observational study' OR 'observational study as topic' OR 'cohort analysis'/exp OR 'analysis, cohort' OR 'cohort analysis' OR 'cohort fertility' OR 'cohort life cycle' OR 'cohort studies' OR 'cohort study' OR 'fertility, cohort').	
Cochrane Library (CENTRAL)	"acute coronary syndrome" OR "myocardial infarction" OR "unstable angina" OR "pulmonary embolism" OR "venous thrombosis" OR "deep venous thrombosis" in Title Abstract Keyword AND "low molecular weight heparin" OR "enoxaparin" OR "unfractioned heparin" OR "heparin" OR "fondaparinux" OR "bivalirudin" in Title Abstract Keyword AND "obesity" OR "abdominal obesity" OR "overweight"	63
LILACS and SciELO	("Acute coronary syndrome" OR "Myocardial Infarction" OR "Heart Attack" OR "Cardiac Attack" OR "Angina, Unstable" OR "Angina, Preinfarction" OR "Myocardial Preinfarction Syndrome" OR "Angina at Rest" OR "Pulmonary Embolism" OR "Pulmonary Thromboembolism" OR "Thromboembolism, Pulmonary" OR "Venous Thrombosis" OR "Phlebothrombosis" OR "Thrombosis, Venous" OR "Deep Vein Thrombosis" OR "Thrombosis, Deep Vein") AND ("Heparin, Low-Molecular-Weight" OR "LMWH" OR "Low-Molecular-Weight Heparin" OR "Enoxaparin" OR "Heparin" OR "Heparinic Acid" OR "alpha-Heparin" OR "fondaparinux" OR "bivalirudin") AND ("Obesity" OR "Obesity, Abdominal" OR "Central Obesity" OR "Overweight")	9 (7 LILACS e 2 SciELO)

SUPPLEMENTAL RESULTS

Anticoagulation strategies used

Of the 6 studies, 5 used unfractionated heparin (UFH), 4 used enoxaparin, and only 1 used fondaparinux. No articles were found that used bivalirudin. For the treatment of ACS, in Spinler et al[1], patients were randomized in the ESSENCE trial to receive 1mg/kg of enoxaparin subcutaneously at 12-hour intervals + placebo bolus and intravenous infusion; or subcutaneous placebo + bolus intravenous UFH 5000 units followed by a continuous heparin infusion adjusted to the aPTT (target = 55-85s). In the TIMI11B trial, participants were randomized to receive a intravenous bolus of UFH of 70U/kg followed by an initial infusion of 15U x kg-1 x h-1 with adjustments to target an aPTT of 1.5-2.5 times the normal value + subcutaneous placebo injections; or enoxaparin with intravenous bolus of 30mg followed by 1mg/kg subcutaneously at 12-hour intervals + intravenous placebo. In both studies, there was no maximum dose for obese patients.

In Mahaffey et al[2], the SYNERGY trial was randomized between intravenous UFH with a bolus of 60U/kg followed by a continuous infusion of 12U/kg/h, adjusted to the target aPPT of 1.5-2.0 times the upper limit of normality of the institution; or enoxaparin 1mg/kg subcutaneously at 12-hour intervals. There was no dose limit. Spinler et al[3], on the other hand, as a retrospective cohort, selected those patients from the CRUSADE initiative who had used enoxaparin for the treatment of ACS and analyzed the average dose given to patients, adopting as an ideal dose a value between 0.95-1.05mg/kg subcutaneously.

For the treatment of VTE, Davidson et al[4] used data from the MATISSE trials. The MATISSE-DVT trial randomized patients to receive fondaparinux with a daily subcutaneous dose of 7.5mg (5mg for <50kg and 10mg for >100kg) + subcutaneous placebo at 12-hour intervals; or subcutaneous enoxaparin 1mg/kg subcutaneously at 12-hour intervals + daily dose of placebo for the treatment of patients with DVT. In the MATISSE-PE trial, for the treatment of PE, patients were also randomized to receive fondaparinux, with the same dosage as the previous study, but alternatively, patients received intravenous UFH with an initial bolus of 5000 units followed by continuous infusion of up to 1250U/h, targeting the aPTT between 1.5-2.5 times the normal value.

The Hosch et al[5] and Shlensky et al[6] studies are retrospective cohorts that evaluated the use of UFH for the treatment of DVT. In Hosch et al[5], the included patients were those who received UFH according to the pharmacy dosing protocol, which consisted of an initial bolus of 80U/kg (maximum dose of 10000 units), followed by a

continuous infusion of 18U/kg/h (maximum dose of 2500U/h), aiming to maintain the aPTT between 57-96s. According to this protocol, the doses are based on body weight, unless the patient exceeds >20% of their Ideal Body Weight (IBW), in which cases the doses are based on Dosing Body Weight (DBW). The definitions of IBW and DBW adopted in the article are in Supplemental Table 5. In Shlensky et al[6], on the other hand, the selected patients were those anticoagulated according to the high-intensity heparin nomogram (HIHN). In these cases, patients received an optional initial bolus of 80U/kg, followed by a continuous infusion of 18U/kg/h (adjusted for aPTT) with doses calculated using the patient's body weight value, regardless of their weight.

SUPPLEMENTAL TABLES

Supplemental table 1: Definitions of major bleeding in each study

Study	Major Bleeding Definition
Spinler 2003[1]	Bleeding resulting in death; retroperitoneal, intracranial, or intraocular bleeding; a decrease in hemoglobin concentration ≥3 g/dL or the need for transfusion ≥2 units of blood.
Davidson 2007[4]	Clinically evident bleeding associated with a decrease of 2g/dL (or 20 g/L) or more in hemoglobin level, leading to transfusion of 2 or more units of red blood cells or whole blood, was retroperitoneal or intracranial, occurred in a critical organ, or contributed to death.
Spinler 2009[3]	Any intracranial bleeding, retroperitoneal bleeding, baseline hematocrit of 28% or higher with red blood cell concentrate transfusion or less than 28% with red blood cell concentrate transfusion and a witnessed bleeding event, or an absolute decrease in hematocrit of at least 12%.
Mahaffey 2010[2]	(TIMI classification)<br><br>Bleeding will be classified as major if associated with a decrease of 5 g/dL in hemoglobin (each unit of red blood cells or whole blood transfused will count as 1 g of hemoglobin) or an absolute decrease of 15% in hematocrit (each unit of red blood cells or whole blood transfused will count as 3% points) or if it is intracranial (confirmed by magnetic resonance imaging or computed tomography).
Hosch 2017[5]	A drop in hemoglobin of ≥2 g/dL from baseline with subsequent transfusion of 2 or more units of packed red blood cells during heparin therapy.
Shlensky 2020[6]	Any fatal bleeding and/or symptomatic bleeding in a critical area or organ, such as intracranial, intraspinal, intraocular, retroperitoneal, intra-articular or pericardial, or intramuscular with compartment syndrome and/or bleeding causing a decrease in hemoglobin level of 2 g/dL or more, or leading to transfusion of ⩾2 units of whole blood or red blood cells.

Supplemental table 2: Summary of the quality evaluation of evidence using the GRADE approach

Clinical Outcome	Number of Studies	Study Design	Quality assessment					RR (CI 95%)	Quality
			Risk of bias	Inconsistency	Indirectness	Imprecision	Publication bias		
Major bleeding	6	RCT[1,2,4] and observational[3,5,6]	-1	not serious	not serious	not serious	not serious	0.90 (0.77-1.04)	LOW*
Mortality	2	RCT[1,2]	-1	not serious	not serious	not serious	not serious	0.71 (0.59-0.87)	MODERATE

RR = Relative Risk; CI = Confidence Interval; RCT = Randomized Clinical Trials.

* For the outcome of major bleeding, we initially judged the evidence as moderate, as it was derived from randomized and observational studies, with the heaviest observational study based on the CRUSADE registry (Spinler 2009[3]) and receiving a maximum score on the NOS.

Supplemental table 3: Characteristics that reduced the level of evidence by GRADE

Domain	Major Bleeding	Mortality
Risk of bias	The studies presented methodological limitations based on the Cochrane Risk of Bias Tool and NOS.	The studies presented methodological limitations based on the Cochrane Risk of Bias Tool and NOS.
Inconsistency	CI mostly overlap<br>Effect estimates are mostly similar<br>I^2 34%	IC mostly overlap<br>Effect estimates are mostly similar<br>I^2 0%
Indirect Evidence	Population, exposure, comparator, and outcomes of each individual study are	Population, exposure, comparator, and outcomes of each individual study are

	consistent with the question of this meta-analysis.	consistent with the question of this meta-analysis.
Imprecision	Narrow confidence interval and a large number of events.	Narrow confidence interval and a large number of events.
Publication bias	"Comprehensive search strategy" and "Symmetric funnel plot".	"Comprehensive search strategy" and "Symmetric funnel plot"

NOS = New-castle Ottawa Scale; CI = Confidence interval

Supplemental table 4: Characteristics that increase the level of evidence according to GRADE.

Domain	Major Bleeding	Mortality
Large effect size	The results are not consistent with a large effect size	A large effect size was not observed
Dose-response gradient	Dose-response gradient was not observed.	Dose-response gradient was not observed.
Residual confounding factors	Residual confounding factors were not identified	Residual confounding factors were not identified

Supplemental table 5: Definition of Ideal Body Weight and Dosing Body Weight[5]

Dosage definitions	
IBW (kg)	Males: 50 + (2.3 x Height in inches > 60 inches)<br>Females: 45.5 + (2.3 x Height in inches > 60 inches)
DBW (kg)	IBW + [0.4 (ABW – IBW)

Abbreviations: ABW: Actual Body Weight; DBW: Dosing Body Weight; IBW: Ideal Borporal Weight.

SUPPLEMENTAL FIGURES

Supplemental Figure 1 – Funnel plots for publication bias per clinical outcome

Supplemental Figure 1.A – Funnel plot for major bleeding

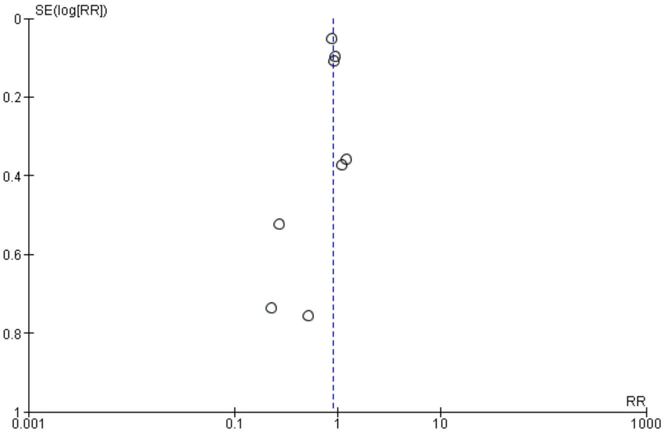

Supplemental Figure 1.B – Funnel plot for mortality

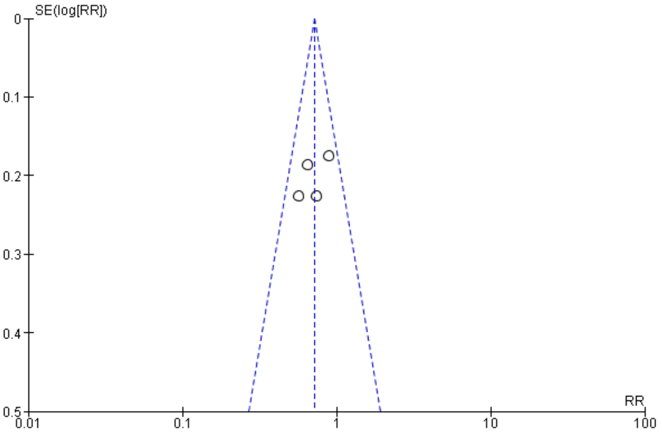

Supplemental Figure 2: Forest plot for major bleeding in obese versus non-obese on ACS studies

Study or Subgroup	Obese Events	Obese Total	Nonobese Events	Nonobese Total	Weight	Risk Ratio M-H, Random, 95% CI	Year
Spinler 2003 (Enoxaparin)	4	921	41	2595	1.7%	0.27 [0.10, 0.77]	2003
Spinler 2003 (UFH)	11	918	25	2563	3.4%	1.23 [0.61, 2.49]	2003
Spinler 2009 (Enoxaparin)	513	7428	900	11633	44.2%	0.89 [0.80, 0.99]	2009
Mahaffey 2010 (Enoxaparin)	138	1585	308	3331	27.0%	0.94 [0.78, 1.14]	2010
Mahaffey 2010 (UFH)	111	1552	257	3369	23.7%	0.94 [0.76, 1.16]	2010
**Total (95% CI)**		**12404**		**23491**	**100.0%**	**0.91 [0.79, 1.04]**	
Total events	777		1531				

Heterogeneity: Tau² = 0.01; Chi² = 6.28, df = 4 (P = 0.18); I² = 36%

Test for overall effect: Z = 1.41 (P = 0.16)

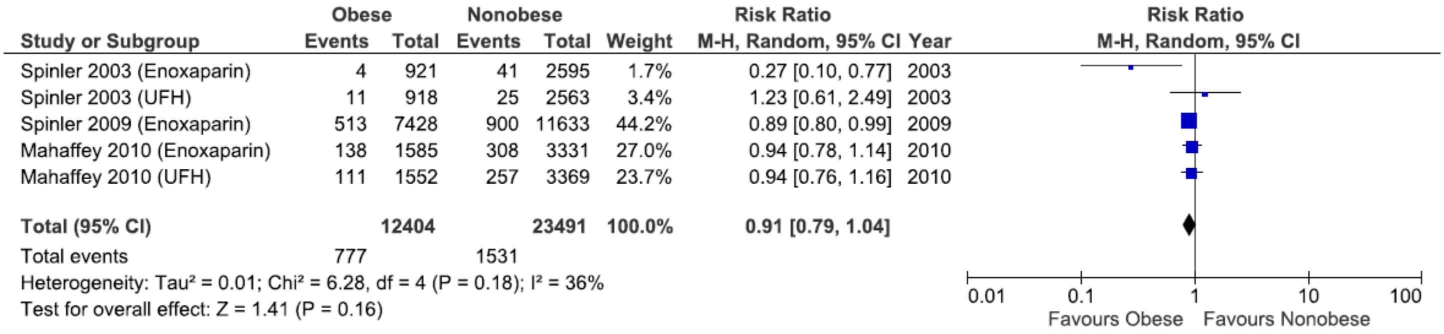

Supplemental Figure 3: Forest plot for major bleeding in obese versus non-obese on VTE studies

Study or Subgroup	Obese Events	Obese Total	Nonobese Events	Nonobese Total	Weight	Risk Ratio M-H, Random, 95% CI	Year
Davidson 2007 (Fondaparinux)	2	590	23	1549	26.9%	0.23 [0.05, 0.97]	2007
Hosch 2017 (UFH)	3	173	4	121	26.1%	0.52 [0.12, 2.30]	2017
Shlensky 2020 (UFH)	13	193	14	230	47.0%	1.11 [0.53, 2.30]	2020
**Total (95% CI)**		**956**		**1900**	**100.0%**	**0.60 [0.22, 1.60]**	
Total events	18		41				

Heterogeneity: Tau² = 0.40; Chi² = 4.16, df = 2 (P = 0.12); I² = 52%
Test for overall effect: Z = 1.03 (P = 0.30)

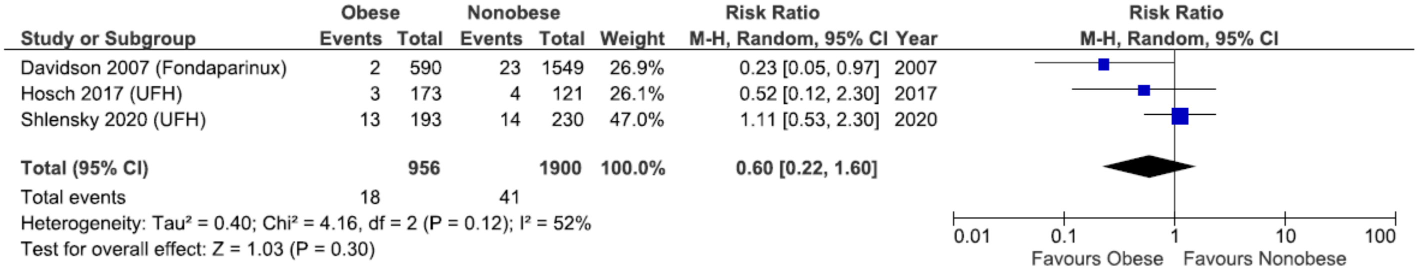

Supplemental Figure 4: Forest plot for major bleeding in obese versus non-obese on enoxaparin studies

Study or Subgroup	Obese Events	Obese Total	Nonobese Events	Nonobese Total	Weight	Risk Ratio M-H, Random, 95% CI	Year
Spinler 2003 (Enoxaparin)	4	921	41	2595	4.3%	0.27 [0.10, 0.77]	2003
Spinler 2009 (Enoxaparin)	513	7428	900	11633	53.9%	0.89 [0.80, 0.99]	2009
Mahaffey 2010 (Enoxaparin)	138	1585	308	3331	41.8%	0.94 [0.78, 1.14]	2010
**Total (95% CI)**		**9934**		**17559**	**100.0%**	**0.87 [0.70, 1.08]**	
Total events	655		1249				

Heterogeneity: Tau² = 0.02; Chi² = 5.40, df = 2 (P = 0.07); I² = 63%
Test for overall effect: Z = 1.26 (P = 0.21)

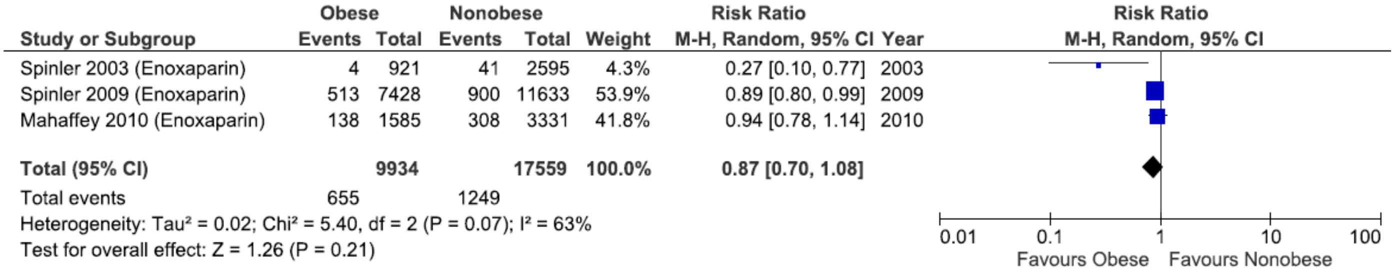

Supplemental Figure 5: Forest plot for major bleeding in obese versus non-obese on UFH studies

Study or Subgroup	Obese Events	Obese Total	Nonobese Events	Nonobese Total	Weight	Risk Ratio M-H, Random, 95% CI	Year
Spinler 2003 (UFH)	11	918	25	2563	7.7%	1.23 [0.61, 2.49]	2003
Mahaffey 2010 (UFH)	111	1552	257	3369	83.4%	0.94 [0.76, 1.16]	2010
Hosch 2017 (UFH)	3	173	4	121	1.8%	0.52 [0.12, 2.30]	2017
Shlensky 2020 (UFH)	13	193	14	230	7.2%	1.11 [0.53, 2.30]	2020
**Total (95% CI)**		**2836**		**6283**	**100.0%**	**0.96 [0.79, 1.17]**	
Total events	138		300				

Heterogeneity: Tau² = 0.00; Chi² = 1.30, df = 3 (P = 0.73); I² = 0%
Test for overall effect: Z = 0.42 (P = 0.67)

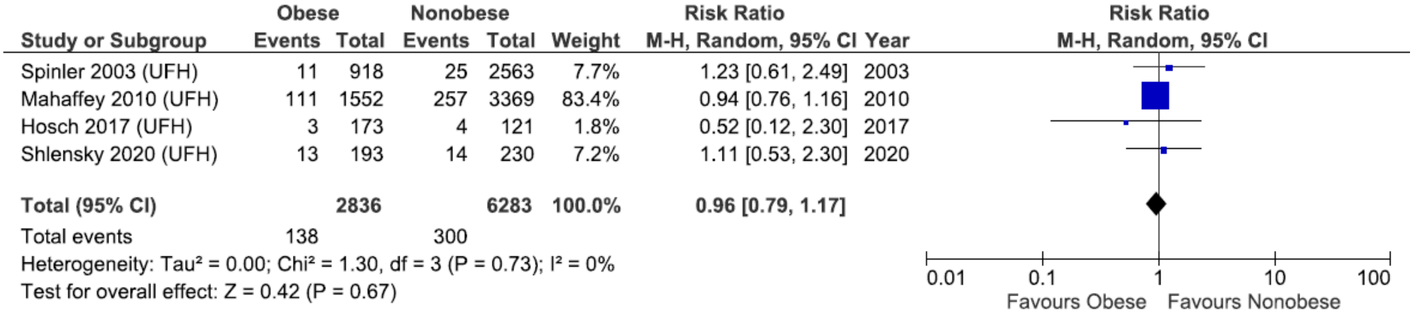

Supplemental Figure 6: Forest plot for major bleeding in obese versus non-obese on Randomized Clinical Trials.

Study or Subgroup	Obese Events	Obese Total	Nonobese Events	Nonobese Total	Weight	Risk Ratio M-H, Random, 95% CI	Year
Spinler 2003 (Enoxaparin)	4	921	41	2595	7.3%	0.27 [0.10, 0.77]	2003
Spinler 2003 (UFH)	11	918	25	2563	13.1%	1.23 [0.61, 2.49]	2003
Davidson 2007 (Fondaparinux)	2	590	23	1549	4.0%	0.23 [0.05, 0.97]	2007
Mahaffey 2010 (Enoxaparin)	138	1585	308	3331	38.5%	0.94 [0.78, 1.14]	2010
Mahaffey 2010 (UFH)	111	1552	257	3369	37.1%	0.94 [0.76, 1.16]	2010
**Total (95% CI)**		**5566**		**13407**	**100.0%**	**0.84 [0.62, 1.14]**	
Total events	266		654				

Heterogeneity: Tau² = 0.05; Chi² = 9.81, df = 4 (P = 0.04); I² = 59%
Test for overall effect: Z = 1.13 (P = 0.26)

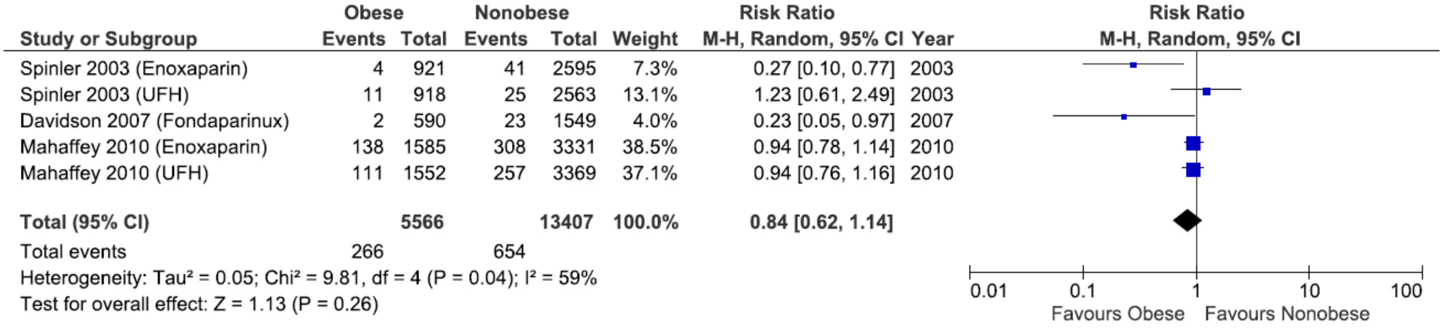

REFERENCES

1. Spinler SA, Inverso SM, Cohen M, Goodman SG, Stringer KA, Antman EM. Safety and efficacy of unfractionated heparin versus enoxaparin in patients who are obese and patients with severe renal impairment: Analysis from the ESSENCE and TIMI 11b studies. Am Heart J. 2003;146(1):33–41.

2. Mahaffey KW, Tonev ST, Spinler SA, Levine GN, Gallo R, Ducas J, et al. Obesity in patients with non-ST-segment elevation acute coronary syndromes: Results from the SYNERGY trial. Int J Cardiol [Internet]. 2010;139(2):123–33. Available from: http://dx.doi.org/10.1016/j.ijcard.2008.10.008

3. Spinler SA, Ou FS, Roe MT, Gibler WB, Ohman M, Pollack C V., et al. Weight-based dosing of enoxaparin in obese patients with non-ST-segment elevation acute coronary syndromes: Results from the CRUSADE initiative. Pharmacotherapy. 2009;29(6):631–8.

4. Davidson BL, Büller HR, Decousus H, Gallus A, Gent M, Piovella F, et al. Effect of obesity on outcomes after fondaparinux, enoxaparin, or heparin treatment for acute venous thromboembolism in the Matisse trials. Journal of Thrombosis and Haemostasis. 2007;5(6):1191–4.

5. Hosch LM, Breedlove EY, Scono LE, Knoderer CA. Evaluation of an Unfractionated Heparin Pharmacy Dosing Protocol for the Treatment of Venous Thromboembolism in Nonobese, Obese, and Severely Obese Patients. Annals of Pharmacotherapy [Internet]. 2017;51(9):768–73. Available from: https://www.embase.com/search/results?subaction=viewrecord&id=L617801831&from=export

6. Shlensky JA, Thurber KM, O'Meara JG, Ou NN, Osborn JL, Dierkhising RA, et al. Unfractionated heparin infusion for treatment of venous thromboembolism based on actual body weight without dose capping. Vascular Medicine (United Kingdom). 2020;25(1):47–54.
