# Supplementary material for: Influência da Obesidade na Segurança e Eficácia do Tratamento Antitrombótico: Uma Revisão Sistemática e Metanálise
Source: Arq Bras Cardiol. 2025 May 12;122(4):e20240544. [Article in Portuguese] doi: 10.36660/abc.20240544 (PMC12108139; doi:10.36660/abc.20240544)
Supplement: Translation

# Translation: Influence of Obesity on the Safety and Efficacy of Antithrombotic Therapy: A Systematic Review and Meta-Analysis

**DOI:** 10.36660/abc.20240544i

**Authors:** Beatriz Rocha Darzé, Queila Oliveira Borges, Mateus S. Viana, Eduardo Sahade Darzé, Luiz Eduardo Fonteles Ritt

**Affiliations:** 1 Brazil Escola Bahiana de Medicina e Saúde Pública, Salvador, BA – Brazil; 2 Brazil D’Or Research Institute, IDOR, Hospital Cardio Pulmonar, Salvador, BA – Brazil

**Keywords:** Acute Coronary Syndrome, Fibrinolytic Agents, Obesity, Prognosis, Venous Thromboembolism

## Abstract

**Background:**

Obese individuals have been historically underrepresented in clinical trials. Considering their association with a higher risk of venous thromboembolism (VTE) and acute coronary syndrome (ACS), it is necessary to establish a more suitable anticoagulation regimen for this group of patients.

**Objectives:**

To evaluate the influence of obesity on the safety and efficacy of antithrombotic therapy in patients with ACS or VTE.

**Methods:**

This is a systematic review and meta-analysis that used 5 main international databases. We selected clinical trials or observational studies that compared the occurrence of clinical outcomes (mortality or bleeding) between obese and non-obese patients using parenteral anticoagulants for the treatment of ACS or VTE. P value < 0.05 was used for all analyses.

**Results:**

Six articles, with a total of 40,939 patients, were eligible, being 3 randomized clinical trials and 3 retrospective cohorts. Of the patients, 87.7% had ACS. The incidence of major bleeding was similar between groups (relative risk [RR]: 0.90, 95% confidence interval [CI]: 0.77 to 1.04, p = 0.14). The outcome remained comparable when studies were analyzed separately by anticoagulant: enoxaparin (RR: 0.87, 95% CI, 0.70 to 1.08, p = 0.21) or unfractionated heparin (RR: 0.96, 95% CI, 0.79 to 1.17, p = 0.67). The mortality rate was measured in only 2 studies, both in ACS, and it was lower in obese patients (RR: 0.71, 95% CI 0.59 to 0.87, p = 0.0007).

**Conclusion:**

In patients treated for VTE or ACS, rates of bleeding were comparable between obese and non-obese patients, regardless of the anticoagulant used. The lower mortality rate observed in obese patients may represent the effect of unaccounted confounding.

## Introduction

Obesity is a chronic disease, reaching epidemic levels.^1^ According to the World Health Organization, the prevalence of obesity has nearly tripled in the last 50 years.^2^

Antithrombotic therapy is a pillar on the treatment of acute coronary syndrome (ACS) and venous thromboembolism (VTE). It significantly reduces the occurrence of ischemic events, but it increases the risk of bleeding, which is related to a higher incidence of death, acute myocardial infarction, and stroke.^3^ Parenteral anticoagulants are administered with dose titration based on patients’ weight,^4^ and the maximum dose limit for obese patients is not well-studied. A study with enoxaparin demonstrated that the obese population has a higher incidence of underdosing, and, among those who receive the recommended dose, obese patients have a higher risk of bleeding.^5^

On the other hand, fondaparinux, when used for the treatment of ACS, is used in a fixed dose.^4^ The OASIS-5 and OASIS-6 studies showed that fondaparinux is non-inferior to enoxaparin for the composite of ischemic events, and it is associated with lower mortality probably due to the reduction in hemorrhagic events.^6,7^ However, since the obese population was not properly represented in these studies, the application of these results to patients with body mass index (BMI) ≥ 30 kg/m^2^ is uncertain.

Excess body fat can still affect the pharmacokinetics and pharmacodynamics of medications,^8^ which, combined with the fact that the obese population is historically underrepresented in clinical trials, leads to uncertainties regarding the real effect of obesity on the efficacy and safety of antithrombotic treatment.

The objective of this study was to evaluate the association between obesity and the occurrence of clinical outcomes among patients treated with parenteral anticoagulation for ACS or VTE.

## Methods

### Study design

This is a systematic literature review with meta-analysis.

### Search strategy

MEDLINE/PubMed, SciELO, EMBASE, Lilacs, and Cochrane Library (CENTRAL), were systematically searched between March 9, 2021 and March 12, 2021. The identification of studies was based on the PECO search strategy, which stands for population (P), exposure (E), comparison (C), and outcomes (O), which were defined as follows:

P: Hospitalized adults using parenteral anticoagulants

(unfractionated heparin [UFH], low molecularweight heparin [LMWH], fondaparinux, or bivalirudin) for the treatment of ACS or VTE.

E: Obesity (≥ 30 kg/m^2^)

C: Non-obesity (< 30 kg/m^2^)

O: Major adverse cardiac events (MACE) or bleedin

PubMed, MeSH, and DeCS tools were used as appropriate for each database. Details of the search strategies performed for each database are available in the Supplemental Methods.

In addition, a manual search was performed on the selected studies’ bibliographic references.

### Inclusion criteria

#### Study types

Original studies published in any year, with full text in English, Portuguese, or Spanish were included. Only articles characterized as randomized clinical trials, prospective cohort studies, retrospective cohort studies, and case-control studies were included.

#### Population

We selected articles that included hospitalized patients with a diagnosis of ACS (unstable angina, non-ST-segment elevation myocardial infarction [NSTEMI], and ST-segment elevation myocardial infarction [STEMI]) or VTE treated with parenteral anticoagulant therapy (UFH, LMWH, fondaparinux, or bivalirudin), considering obesity as exposure and non-obesity as comparator.

#### Clinical outcomes

Clinical outcomes included occurrence of MACE (defined as death, myocardial infarction, or stroke) or major bleeding. The definition of each outcome for our study was based on the definitions adopted by the included articles. The definitions of major bleeding adopted in each study are available in Supplemental Table 1.

**Table 1:** – General characteristics of selected studies and their populations

Studies	Country	Study design	Total duration of the study	Anticoagulant indication	Anticoagulant used	Eligibility criteria	Definition of exposure	Sample size	Obese, n (%)	Male, n (%)
*Spinler 2003*^9^	USA	Post-hoc analysis of RCT (ESSENCE and TIMI 11B)	ESSENCE: October 1994 to May 1996 TIMI 11B: August 1996 to March 1998	ACS	ESSENCE: enoxaparin or UFH TIMI 11B: enoxaparin or UFH	Patients with NSTEACS who received continuous IV enoxaparin or UFH. Those with CrCl < 30 mL/min (ESSENCE) or serum creatinine ≥ 2.0 mg/dL (TIMI 11B) were excluded from the trials.	Obesity was defined as BMI ≥ 30 kg/m^2^.	6997	1839 (26.3%)	4593 (65.6%)
*Davidson 2007*^11^	USA	Post-hoc analysis of RCT (MATISSE-DVT and MATISSE-PE)	MATISSE-DVT: April 2000 to July 2001 MATISSE-PE: May 2000 to March 2002	VTE	MATISSE-DVT: fondaparinux or enoxaparin MATISSE-PE: fondaparinux or UFH	Data were included for patients who received at least 1 dose of study drug (for the treatment of VTE) and who had available results for the primary study endpoints (VTE recurrence or bleeding).	Obesity was defined as BMI ≥ 30 kg/m^2^.	4327	1216 (28.1%)	2091 (48.3%)
*Spinler 2009*^5^	USA	Observational retrospective cohort study	January 2004 to March 2006	ACS	Enoxaparina	Patients enrolled in CRUSADE between January 1, 2004 and March 31, 2006, who received enoxaparin for the initial treatment of NSTEACS and for whom information on the initial dose of enoxaparin was collected. Patients who were transferred to another institution, did not have documented BMI, did not have documented estimated CrCl or had CrCl < 30 ml/min, or underwent coronary artery bypass surgery during hospitalization were excluded.	Patients were divided into 4 groups based on BMI: < 18.5; 18.5 to 24.9; 25.0 to 29.9; ≥ 30 kg/m^2^.	19061	7428 (39%)	11613 (60.9%)
*Mahaffey 2010*^10^	USA	Post-hoc analysis of RCT (SYNERGY)	SYNERGY: August 2001 to December 2003	ACS	SYNERGY: enoxaparin or UFH	Patients with NSTEACS. Patients were excluded if they had contraindications for UFH or enoxaparin, underwent PCI or thrombolytic therapy within the previous 24 hours, were at high risk of bleeding complications due to recent stroke or surgery, had INR > 1.5, had past or present bleeding disorder, or had CrCl < 30 mL/min.	Patients were divided into 5 groups based on BMI: < 20; 20 to 25; 25 to 30; 30 to 35; ≥ 35 kg/m^2^.	9837	3137 (31.9%)	6514 (66.2%)
*Hosch 2017*^12^	USA	Observational retrospective cohort study	July 2013 to July 2015	VTE	UFH	Patients ≥ 18 years old who received UFH for the treatment of VTE (DVT and/or PE), managed by pharmacy dosing protocol. Patients who obtained a therapeutic aPTT less than 6 hours after starting heparin were excluded.	Patients were divided into 3 groups based on BMI: < 30; 30 to 40; ≥ 40 kg/m^2^.	294	173 (58.8%)	143 (48.6%)
*Shlensky 2020*^13^	USA	Observational retrospective cohort study	January 2010 to December 2016	VTE	UFH	Patients ≥18 years of age who had documented VTE as an indication for heparin and had been in the HIHN for at least 24 hours. Patients who did not consent to review of their medical records for research, received a thrombectomy (due to increased risk of bleeding), or received a fibrinolytic agent were excluded.	Patients were divided into 3 groups based on BMI: < 30; 30 to 40; ≥ 40 kg/m^2^.	423	193 (45.6%)	223 (52.7%)

ACS: acute coronary syndrome; aPTT: activated partial thromboplastin time; BMI: body mass index; CrCl: creatinine clearance; DVT: deep vein thrombosis; HIHN: high-intensity heparin nomogram; INR: international normalized ratio; IV: intravenous; NSTEACS: non-ST elevation acute coronary syndrome; PCI: percutaneous coronary intervention; PE: pulmonary embolism; UFH: unfractionated heparin; VTE: venous thromboembolism. The significance level adopted by all studies was 5%.

## Exclusion criteria

### Study types

Case reports, case series, literature reviews, and systematic reviews were excluded. Prevention/prophylaxis studies were also excluded.

### Population

Studies that included patients under the age of 18 and adults with creatinine clearance < 10 mL/min were excluded.

## Identification and selection of studies

Two authors independently performed the search, selection, and application of eligibility criteria.

Based on the results of the searches conducted on the databases, the selection process was carried out individually in three stages: (1) removal of duplicates; (2) exclusion of articles that did not meet the eligibility criteria based on the title and abstract; (3) and full-text reading of the selected articles, with a new application of eligibility criteria to identify their quality and relevance to the proposed objective. Any disagreements between the authors were resolved through discussion and dialogue in the presence of a third author.

The Rayyan QCRI tool was used for article selection, and both researchers were blinded to each other’s decisions throughout the process.

## Data extraction

The following characteristics of the studies were extracted: title, reference, type of study (randomized clinical trial or observational study), country and year of publication, and sample size. The following data were also collected on the participants of each study: number of participants, age, number of male and female participants, number of obese and non-obese participants, mean BMI, type of ACS (STEMI, NSTEMI, or unstable angina) and VTE (deep vein thrombosis or pulmonary embolism), and antithrombotic strategy performed.

All outcomes evaluated by the studies were extracted, with a focus on clinical outcomes and comparison between obese and non-obese individuals.

The data extraction process was performed independently by two authors, and discrepancies were resolved by discussion with a third author. Review Manager (Revman), version 5.4 software was used for data recording, as well as data extraction and management.

## Risk of bias analysis

The assessment of the risk of bias included the evaluation of the methods of randomization, treatment allocation, blinding, selection and comparability of study groups, and outcome assessment, which was performed at the study level. The following tools were used: the Newcastle-Ottawa Scale (NOS) for observational studies and the Cochrane Risk of Bias Tool (RoB) for randomized clinical trials. These evaluations were performed by two independent authors, and discrepancies were resolved by discussion with a third author.

Publication bias was assessed through visual inspection of funnel plots corresponding to the meta-analyses of primary outcomes, which were generated using RevMan 5.4 software.

## Quality of evidence analysis

To assess the quality of evidence, the GRADE methodology was applied to each outcome studied. This evaluation was performed by two independent authors, and discrepancies were resolved by discussion with a third author.

## Statistical analysis

For comparison of primary outcomes between the obese and non-obese groups, Relative risk (RR) and 95% confidence intervals (CI) were used as analytical parameters. For each antithrombotic group (enoxaparin, UFH, fondaparinux), RR and 95% CI were generated from the absolute number of patients and outcomes. The statistical analysis and forest plots were performed using RevMan 5.4 software.

Heterogeneity was assessed by visual inspection of the forest plot (analysis), along with consideration of the chi-square test for heterogeneity and the I^2^ statistic (Higgins test). Heterogeneity was considered statistically significant with p < 0.1 or by the heterogeneity percentage, which was classified as low (25% or less), moderate (26% to 50%), or high (greater than 50%). Studies were also examined for methodological and clinical heterogeneity, particularly if statistically significant heterogeneity was identified.

Results from comparable groups of studies were pooled using a random effects model.

P values < 0.05 were considered significant for all analyses.

## Subgroup analysis

Studies were divided into subgroups for sensitivity analysis. Subgroup analysis was performed by type of medication used in the selected studies (separating groups into LMWH/enoxaparin, UFH, fondaparinux, and bivalirudin) and by treatment indication (ACS or VTE). We also performed a sensitivity analysis including only randomized clinical trials.

## Ethical considerations

As this was a systematic review, it was not necessary to submit the study to the Research Ethics Committee. The study protocol was previously published on the PROSPERO platform on April 30, 2021 (CRD42021243189). The study protocol is available at https://www.crd.york.ac.uk/prospero/display_record.php?RecordID=243189.

## Results

### Identification and selection of studies

The database search identified 726 records, of which 97 were duplicates, leaving 629. No eligible studies were found through manual search. After the screening process with title and abstract, 20 studies were selected for full reading, of which 14 were excluded based on eligibility criteria. At the end of the selection process, 6 studies were included for qualitative analysis, as well as quantitative analysis (meta-analysis), as shown in Figure 1.

**Figure 1:** – PRISMA flow diagram (article selection). HIT: heparin-induced thrombocytopenia.

### Characteristics of included studies

The excluded studies with their respective exclusion criteria were presented in Figure 1. In the end, 6 studies were selected for the systematic review, of which 3 were retrospective cohorts and 3 were post-hoc analyses of randomized clinical trials (Table 1). Spinler et al.^9^ performed a subgroup analysis based on the combined data of the ESSENCE and TIMI 11B clinical trials, while Mahaffey et al.^10^ used the SYNERGY trial database. The 3 randomized studies compared enoxaparin to UFH for the treatment of ACS. Davidson et al.^11^ used data from the MATISSE clinical trials, which tested fondaparinux for the treatment of VTE, as an alternative to classic heparin treatment (UFH, enoxaparin). Details of the anticoagulant strategies adopted in each study are available in the Supplemental Results.

A total of 40939 patients who received anticoagulation for the treatment of ACS or VTE, between 1994 and 2016, were included in the review, of which 35895 (87.7%) presented with ACS. In only 1 study,^12^ the obese group represented more than half of the studied population (58.8%), while, in general, this prevalence was 34.2%. The prevalence of the male sex was 61.5%. All studies were based in the United States; however, patients were recruited from different regions of the world such as Europe, North America, South America, and Oceania. The eligibility criteria and definition of exposure of each included study can be observed in Table 1.

### Risk of bias in the included studies

Studies characterized as post-hoc analyses of randomized clinical trials presented methodological weaknesses related to known allocation and other biases, as seen in Figures 2A and 2B. In Davidson et al.^11^ and Mahaffey et al.,^10^ there was no blinding of participants and evaluators regarding the type of intervention applied, justified by the different routes of administration and dosage of medications (UFH by intravenous route; enoxaparin and fondaparinux by subcutaneous route). Spinler et al.^9^ and Davidson et al.,^11^ on the other hand, obtained their analyses through the merger of two randomized clinical trial databases, increasing the risk of bias in the selected sample, due to compromised randomization and adopted methodology.

Regarding the cohort studies, Spinler et al.^5^ was classified as low risk of bias, totaling 9 stars. A limited follow-up period was observed in Shlensky et al.^13^ and Hosch et al.,^12^ and the latter also presented methodological weaknesses related to comparability between groups (Table 2).

**Table 2:** – Risk of bias analysis for the observational studies

Studies	Selection	Comparability	Outcomes
*Spinler 2009*^5^	****	**	***
*Hosch 2017*^12^	****	-	**
*Shlensky 2020*^13^	****	**	**

The analysis of funnel plots in Supplemental Figures 1A and 1B makes it possible to infer a low risk of publication bias for all articles included in the meta-analysis, despite the reduced number of studies included.

### Main findings of the studies

Table 3 shows the incidence of clinical outcomes evaluated by the studies, according to medication used, for the obese and non-obese groups. All studies included in the review assessed the rate of major bleeding during hospitalization, which was 6.0% (95% CI: 5.6% to 6.4%) in obese patients and 6.2% (95% CI: 5.9% to 6.5%) in non-obese patients. The definitions of major bleeding applied in each study are displayed in the Supplemental Results. Regarding efficacy outcomes, only 2 studies analyzed mortality at 30 to 43 days of follow-up. The mortality rate in the obese group was 2.6% (95% CI: 2.2% to 3.0%), and in the non-obese group it was 3.6% (95% CI: 3.3% to 4.0%). Only Spinler et al.^9^ reported recurrent myocardial infarction, while the outcome of stroke was not measured in any of the studies.

**Table 3:** – Clinical outcomes for obese versus non-obese by type of antithrombotic used

Studies	Anticoagulant (indication)	Outcome	Follow-up	Incidence in obese n/N (%)	Incidence in non-obese n/N (%)
*Spinler 2003*^9^	Enoxaparin (ACS)	Death	43 days	24/921 (2.6%)	91/2595 (3.5%)
		AMI	43 days	45/921 (4.9%)	125/2595 (4.8%)
		UR	43 days	83/921 (9%)	257/2595 (9.9%)
		Death/AMI/UR	43 days	132/921 (14.3%)	418/2595 (16.1%)
		Major bleeding	Hospitalization	4/921 (0.4%)	41/2595 (1.6%)
		Any bleeding	Hospitalization	107/921 (11.7%)	243/2595 (9.5%)
	UFH (ACS)	Death	43 days	23/918 (2.5%)	113/2563 (4.4%)
		AMI	43 days	56/918 (6.1%)	154/2563 (6%)
		UR	43 days	107/918 (11.7%)	308/2563 (12%)
		Death/AMI/UR	43 days	165/918 (18.0%)	492/2563 (19.2%)
		Major bleeding	Hospitalization	11/918 (1.2%)	25/2563 (1%)
		Any bleeding	Hospitalization	48/918 (5.3%)	101/2563 (4%)
*Davidson 2007*^11^	Fondaparinux (VTE)	Recurrent VTE	3 months	22/594 (3.7%)	61/1560 (3.9%)
		Major bleeding	Hospitalization	2/590 (0.3%)	23/1549 (1.5%)
	Heparins* (VTE)	Recurrent VTE	3 months	30/622 (4.8%)	70/1551 (4.5%)
		Major bleeding	Hospitalization	7/611 (1.1%)	18/1540 (1.2%)
*Spinler 2009*^5^	Enoxaparin (ACS)	Major bleeding	Hospitalization	513/7428 (6.9%)	900/11.633 (7.7%)
*Mahaffey 2010*^10^	Enoxaparin (ACS)	Death/AMI	30 days	202/1585 (12.8%)	486/3331 (14.5%)
			6 months	262/1585 (16.5%)	605/3331 (18.2%)
		Death	30 days	37/1585 (2.3%)	119/3331 (3.6%)
			6 months	69/1585 (4.4%)	209/3331 (6.3%)
			1 year	108/1585 (6.8%)	269/3331 (8%)
		Severe bleeding (GUSTO)	Hospitalization	39/1585 (2.5%)	94/3331 (2.8%)
		Major bleeding (TIMI)	Hospitalization	138/1585 (8.7%)	308/3331 (9.2%)
	UFH (ACS)	Death/AMI	30 days	206/1552 (13.3%)	515/3369 (15.3%)
			6 months	253/1552 (16.3%)	628/3369 (18.6%)
		Death	30 days	45/1552 (2.9%)	110/3369 (3.3%)
			6 months	75/1552 (4.8%)	182/3369 (5.4%)
			1 year	95/1552 (6.1%)	262/3369 (7.8%)
		Severe bleeding (GUSTO)	Hospitalization	28/1552 (1.8%)	76/3369 (2.3%)
		Major bleeding (TIMI)	Hospitalization	111/1552 (7.2%)	257/3369 (7.6%)
*Hosch 2017*^12^	UFH (VTE)	Bleeding event Hgb drop ≥ 2 g/dL and ≥ 2 units PRBC received	Hospitalization	20/173 (11.5%)	17/121 (14%)
			Hospitalization	3/173 (1.7%)	4/121 (3.3%)
		Thrombotic events	Hospitalization	0/173	0/121
*Shlensky 2020*^13^	UFH (VTE)	Major bleeding	Hospitalization	13/193 (6.7%)	14/230 (6.1%)
		Thrombotic complications	Hospitalization	1/193 (0.5%)	1/230 (0.4%)

ACS: acute coronary syndrome; AMI: acute myocardial infarction; GUSTO: Global Use of Strategies to Open Occluded Arteries; Hgb: hemoglobin; PRBC: packed red blood cells; TIMI: Thrombolysis in Myocardial Infarction; UFH: unfractionated heparin; UR: urgent revascularization; VTE: venous thromboembolism. *UFH in MATISSE-PE and enoxaparin in MATISSE-DVT.

### Summary of results

#### Major bleeding

The results of this analysis showed that the rate of major bleeding during the intra-hospital period was similar between obese and non-obese patients using parenteral anticoagulants, as shown in Figure 3A. The heterogeneity was considered moderate. The incidence of the outcome remained comparable when studies on ACS and VTE were analyzed separately, as well as when analyzed by anticoagulant (enoxaparin or UFH). The rate of bleeding was also similar between groups when analyzing randomized clinical trials separately. The forest plots of subgroup analyses are presented in Supplemental Figures 2 to 6.

#### Mortality

Only 2 articles evaluated the incidence of death, both in the setting of ACS.^9,10^ During a follow-up period of 30 to 43 days, the mortality rate was lower in obese patients compared to non-obese patients, as shown in Figure 3B. The heterogeneity was considered low. It was not possible to perform an individualized analysis for each antithrombotic agent due to the small number of articles in the analysis.

#### Other outcomes

For the other previously established outcomes (myocardial infarction and stroke), meta-analysis was not performed due to the limited number of articles. Only 1 study (Spinler et al.^9^) evaluated the outcome of myocardial infarction alone, while the incidence of stroke was not measured in any of the studies included in the review.

#### Quality of evidence (GRADE)

Supplemental Table 2 provides a summary of the assessment of the quality of evidence using the GRADE approach. For the outcome of major bleeding, the evidence was initially classified as moderate, as it included both randomized and observational studies. After evaluating each domain, we judged the quality of evidence to be low, with a 1-point downgrade in the domain of risk of bias. For the outcome of mortality, the evidence was initially classified as high, as it was derived solely from randomized clinical trials. After a 1-point downgrade in the domain of risk of bias, it was classified as moderate quality. No characteristics were identified to increase the level of evidence. The detailed assessment using the GRADE method can be found in Supplemental Tables 3 and 4.

## Discussion

The present study evaluated the influence of obesity on the safety and efficacy of antithrombotic treatment after acute coronary events or VTE. We found that obesity has little or no impact on the incidence of clinical outcomes after anticoagulant therapy. Few studies reported thrombotic and death outcomes, limiting conclusions about efficacy. On the other hand, regarding bleeding events, the use of UFH, enoxaparin, and fondaparinux seems to be safe in the obese population (BMI ≥ 30 kg/m^2^).

### Acute coronary syndrome

In the context of ACS, in the combined analysis of the ESSENCE and TIMI 11B trials, there was no difference between obese (BMI ≥ 30kg/m^2^) and non-obese patients in the incidence of the composite primary outcome of death, myocardial infarction, and need for urgent revascularization or major bleeding occurrence. However, there was a trend towards a higher risk of any bleeding among those with a higher BMI.^9^ Furthermore, the incidence of major bleeding was similar between obese and non-obese patients treated with UFH, but it favored patients with BMI ≥ 30 kg/m^2^ among those treated with enoxaparin (RR: 0.27, 95% CI: 0.10 to 0.77) (Figure 3A). Data from the SYNERGY trial, in which 9873 patients were randomized to receive UFH or enoxaparin, did not find a significant difference in the occurrence of death, myocardial infarction, and major bleeding associated with BMI.^10^ These findings, confirmed in the present meta-analysis, support the weight-based approach without a dose cap (Figure 3A).

On the other hand, Spinler et al.,^5^ based on the CRUSADE registry, showed that overweight and obese patients tend to be treated with reduced doses of enoxaparin (< 0.95 mg/kg), when compared to those with normal weight. In addition, it was observed that patients weighing > 150 kg in this cohort, although underrepresented (n = 37), who were treated with recommended enoxaparin doses (0.95 to 1.05 mg/kg), had a higher risk of bleeding when compared to those who received a reduced dose. This might explain why this meta-analysis was not able to demonstrate a difference in bleeding rates between obese and non-obese patients. However, the small sample size and observational nature of the study preclude definitive conclusions.

The OASIS-5 study showed that fondaparinux was non-inferior to enoxaparin regarding the occurrence of ischemic events after NSTEMI or unstable angina, and it was associated with a significant reduction in the risk of major bleeding and mortality at 30 and 180 days. However, there was a low representation of the obese population in the study.^6^ Thus, the question about the efficacy of the fixed dose of 2.5 mg in obese patients is still uncertain. In an internal analysis, among patients who used fondaparinux, the incidence of bleeding during therapy and on day 9 was inversely proportional to body weight, and those weighing > 100 kg had a better prognosis,^14^ suggesting that the safety of this treatment may not be affected by the presence of obesity. No studies were found that compared the incidence of clinical outcomes between obese and non-obese patients using fondaparinux.

Regarding the outcome of mortality, the synthesis of the results of the studies by Spinler et al.^9^ and Mahaffey et al.^10^ showed a tendency for a better prognosis among obese patients in the period of 30 to 43 days, a finding previously described in the literature^15-17^ as the “obesity paradox.” The fact that obese patients are commonly younger, less frail, and have fewer risk predictors for bleeding in the moment of hospitalization may justify results related to a lower occurrence of clinical outcomes in this population group. However, the inability to perform statistical adjustments, a characteristic of our study design, makes it difficult to exclude that confounding variables and differential treatment strategies in the obese population may be the reason for the different mortality rate or even for not finding difference regarding bleeding events.

### Venous thromboembolism

Barletta et al. compared the values of activated partial thromboplastin time (aPTT) achieved by patients with and without morbid obesity (BMI > 40 kg/m^2^), using the weight-based nomogram for the treatment of VTE. They showed that those in the higher weight group had a higher incidence of overdose and that BMI was an independent predictor of supratherapeutic aPTT values, suggesting a possible need for a dose limit for those with BMI > 40 kg/m^2^.^18^

In this scenario, two studies in this review aimed to study the influence of BMI on the efficacy and safety of UFH in patients with VTE. In Hosch et al.,^12^ patients received UFH dose based on body weight, unless their weight exceeded 20% of the ideal body weight, in which case it was based on the ideal body weight. In contrast, Shlensky et al.^13^ conducted the study in the scenario where all patients, regardless of weight, received the same dosage of medication based on body weight. Despite the different approaches, neither study found a difference between the three BMI classes (< 30, 30 to 40, and > 40 kg/m^2^) regarding the time required to reach the first therapeutic value of aPTT and the occurrence of bleeding during the study. Supporting the findings of Barletta et al.,^18^ Shlensky et al.^13^ also identified a higher incidence of supratherapeutic aPTT values in the group of morbidly obese patients; however, this finding was not reflected in the occurrence of bleeding, suggesting that there is no clinical impact of BMI on the safety of UFH in VTE. However, in addition to the small sample size, the studies have the limitation of identifying bleeding events through the discharge summary, which may potentially have underestimated the number of events.

Fondaparinux was tested as an alternative option to heparins (UFH and enoxaparin) in the MATISSE clinical trials, which demonstrated the non-inferiority of this medication when used in 3 weight-based doses (5.0, 7.5, and 10 mg).^19,20^ Davidson et al.,^11^ based on MATISSE data, showed that the results were maintained in the subgroup of obese patients (BMI > 30), corroborating the findings of our present study. In our meta-analysis, despite the wide confidence interval, the results showed that obese patients using fondaparinux tended to have a reduced rate of major bleeding compared to non-obese patients (RR: 0.23, 95% CI: 0.05 to 0.97) (Figure 3A). In MATISSE, although there was a weight-based dose adjustment in the administration of fondaparinux (5 mg for < 50kg; 7.5 mg for 50 to 100 kg; 10 mg for > 100 kg), all patients weighing > 100 kg received the same daily dose of 10 mg, without dose progression with weight, which may be a potential bleeding protection factor for morbidly obese patients.

### Limitations

The present review has some limitations. Due to the high prevalence of patients with ACS (87.7%) in the studied sample, this analysis mainly concerns patients who suffered an acute coronary event, with limited conclusions about patients with VTE. The classification of bleeding events and the adopted anticoagulation strategies were diverse, limiting the combination of these data. Due to the variations in outcome definition and collection, it was not possible to perform an analysis of major combined outcomes. Furthermore, due to the inability to control for confounding variables, other factors such as the use of other antiplatelets and anticoagulants and the differences in clinical profile between obese and non-obese patients (age, sex, comorbidities) may have influenced the results obtained. The population also varied between studies. Future studies with meta-regression analysis, which could control for these variables, would be of interest. In addition, the long inclusion period of the studies (2003 to 2020), associated with significant advances in adjuvant treatment of ACS during this period, may have influenced the results. Finally, patients with grade III obesity are still poorly represented, and studies focused on this specific population are necessary.

### Implications for practice and future research

Our results, although hypothesis-generating, suggest that a differential anticoagulant regimen may not be necessary for obese patients with ACS or VTE. Future randomized studies in the specific population of obese patients are necessary to obtain more robust conclusions.

## Conclusions

In patients treated for VTE or ACS, rates of bleeding were comparable between obese and non-obese patients, regardless of the anticoagulant used. The lower mortality rate observed in obese patients may represent the effect of unaccounted confounding. Subsequent studies are needed to validate these findings.

**Figure 2:** – Risk of bias analysis for the clinical trials.

**Figure 3:** – Forest plots. CI: confidence interval; M-H: Mantel-Haenszel analysis; UFH: unfractionated heparin.

## S-1 *Supplemental Materials

For additional information, please click here Writing of the manuscript and Critical revision of the manuscript for content http://abccardiol.org/supplementary-material/2025/12204/2024-0544_AO_Supplemental_Material_obesity_and_antithrombotics.pdf
